# SLA-YOLO: A Lightweight SAR Ship Detection Model

**DOI:** 10.3390/s26196317

**Published:** 2026-10-07

**Authors:** Chaoyue Yin, Nan Bi

**Affiliations:** College of Electrical Engineering, Northeast Electric Power University, Jilin City 132012, China; 19105412801@163.com

**Keywords:** lightweight detection, synthetic aperture radar, ship detection, YOLO11

## Abstract

Reliable ship detection in synthetic aperture radar (SAR) imagery is challenged by speckles, coastal clutter, scale variation, dense berthing, weak target responses, and limited computing resources. SLA-YOLO is a lightweight YOLO11n detector that coordinates established mechanisms across the backbone, neck, and head. LESCNet combines ECA-enhanced depthwise-pointwise convolution with residual large-kernel refinement; C3k2-DAPE applies input-conditioned branch weighting and channel recalibration at selected fusion blocks; and SA-LSCD shares intermediate transformations while retaining scale-specific predictors. On fixed SSDD and HRSID splits, SLA-YOLO achieves mAP@0.5 values of 97.3% and 90.8% and mAP@0.5:0.95 values of 68.2% and 64.3%, respectively, with 1.70 million parameters and 3.6 GFLOPs. Relative to YOLO11n, parameters and FLOPs decrease by 34.1% and 42.9%, while mAP@0.5 increases by 4.4 and 5.2 percentage points, respectively. A complete 2^3^ ablation shows positive average main effects for all three components and context-dependent interactions. Across four no-fine-tuning transfer settings, SLA-YOLO improves mAP@0.5 by 3.5–6.3 points. Three-seed experiments yield 97.17 ± 0.31% and 90.87 ± 0.35% mAP@0.5 on SSDD and HRSID. Small-ship AP improves over YOLO11n by 10.1 ± 0.5 and 6.9 ± 0.7 points, with larger gains inshore. Network-only throughput reaches 66.8 and 63.4 FPS on an RTX 3090; embedded-device performance remains unverified.

## 1. Introduction

Synthetic aperture radar (SAR) is an active microwave imaging technique that can observe wide maritime areas by day or night and under cloud cover, haze, and other adverse weather conditions [1,2]. These properties make SAR an important information source for ocean observation, maritime safety, illegal fishing surveillance, port management, search and rescue, and disaster assessment. Unlike optical sensors, SAR records coherent backscattering rather than reflected visible light. The resulting images contain speckles, sidelobe artifacts, strong responses from coastal structures, and intensity variations associated with incidence angle, polarization, sea state, vessel material, and imaging mode. Ships may therefore appear as bright compact points, elongated scattering structures, fragmented responses, or weak targets surrounded by sea clutter.

Conventional SAR ship detection commonly relies on statistical clutter modeling and constant false-alarm-rate processing. Although such methods are interpretable and computationally convenient, their performance depends strongly on the assumed background distribution and the accuracy of sea–land masking. Coastal scenes, heterogeneous clutter, wake structures, and dense ports can violate these assumptions and produce missed detections or false alarms. Deep learning has therefore become a widely used alternative because classification and bounding-box regression can be optimized jointly from annotated data. Public benchmarks such as the SAR Ship Detection Dataset (SSDD) [3] and the High-Resolution SAR Images Dataset (HRSID) [4] have supported systematic development and evaluation.

Modern object detectors can be broadly categorized into two-stage and one-stage frameworks. Faster R-CNN first generates candidate regions and then performs classification and localization, often providing strong accuracy at considerable computational and memory cost [5]. One-stage methods such as SSD predict objects directly from feature maps and generally provide higher throughput [6]. Feature Pyramid Networks (FPN) and Path Aggregation Networks (PANet) strengthen hierarchical feature use [7,8], while Transformer-based detectors introduce global interaction and end-to-end set prediction [9]. Direct transfer of these methods to SAR imagery remains difficult because its imaging mechanism, target appearance, and clutter statistics differ substantially from those of natural images.

Deployment introduces a second challenge. Maritime monitoring systems may operate on satellites, unmanned platforms, coastal stations, or other edge devices with constrained power, memory, and computing capacity. Lightweight design therefore requires more than replacing standard convolution with depthwise convolution. Depthwise operators reduce arithmetic cost but process channels independently and can weaken cross-channel representation. Moreover, low FLOP counts do not necessarily yield low latency because memory access, network fragmentation, parallel branches, and hardware utilization also affect execution speed [10]. A practical detector must therefore balance representation, localization, parameter count, computation, and measured throughput. In this work, throughput is measured reproducibly on the available RTX 3090 and is interpreted as desktop-GPU network-only performance; embedded-device performance requires separate validation on the target platform.

Recent SAR ship detectors address this trade-off through compact backbones, feature fusion, attention, reparameterization, and Transformer variants. A lightweight Faster R-CNN reduces feature-extraction cost but retains region-proposal overhead [11]. YOLOv5-MNE combines a MobileNet-derived block with ECA and coordinate attention [12], while LMSD-YOLO combines lightweight convolution with multiscale enhancement [13]. Other studies improve regression and feature representation [14], introduce a lightweight vision Transformer [15], or use large kernels and structural reparameterization [16]. LH-YOLO, LSD-DETR, and AC-YOLO further explore compact YOLO or Transformer designs [17,18,19]. Reviews nevertheless identify persistent difficulties in small-target representation, complex coastal clutter, evaluation consistency, and the gap between theoretical complexity and deployment speed [2,20].

Among these detectors, AC-YOLO [19] is the closest comparator because it also adapts YOLO11 to SAR ship detection. AC-YOLO replaces C2PSA with ACmix, which combines self-attention and convolution; its CCFM neck performs cross-scale alignment and channel-aware dynamic processing; and MPDIoU changes bounding-box regression training. These are substantive adaptive mechanisms, so SLA-YOLO does not claim to introduce attention or dynamic fusion to YOLO11 for the first time. These choices leave open the compute-allocation question addressed here: whether lightweight local encoding can serve as an alternative to ACmix’s dual path, where selection belongs within the neck, and which head parameters can be shared. LESCNet combines ELDWConv and residual RSCB as a lightweight spatial-channel encoder intended to retain faint local target structure. C3k2-DAPE weights four alternative transformations within selected neck fusion bottlenecks, rather than using CCFM’s cross-scale alignment and channel-aware dynamic convolution. SA-LSCD shares an intermediate prediction transformation across P3, P4, and P5 but retains separate final class and box predictors. AC-YOLO’s reported architecture does not examine this partial-sharing boundary in the detection head. This identifies a design question left open by AC-YOLO rather than a demonstrated failure; Section 4.5 compares the two reimplemented models under the same training and evaluation configuration.

Three related design trade-offs motivate SLA-YOLO. MobileNet-derived SAR backbones such as YOLOv5-MNE and LMSD-YOLO [12,13] gain efficiency through factorized operations, making the balance between inexpensive spatial filtering and channel interaction important for weak scattering cues. FPN and PANet [7,8] use prescribed fusion routes, whereas SAR methods such as LMSD-YOLO and AC-YOLO [13,19] already introduce adaptive mechanisms; the remaining question is where input-dependent processing should be placed. Finally, LH-YOLO’s shared head [17] shows the value of parameter reuse, while leaving the degree of scale-specific prediction as a design choice. Section 2.1 relates these trade-offs to specific prior methods and to LESCNet, C3k2-DAPE, and SA-LSCD, respectively.

To address these issues, SLA-YOLO redesigns YOLO11n by assigning established mechanisms to three different locations: inexpensive spatial and channel encoding in the backbone, input-conditioned transformation selection at selected neck fusion blocks, and intermediate parameter sharing with scale-specific outputs in the head. The methodological contribution is a constrained detector-level allocation: it specifies where adaptive computation is introduced, which representation is shared across scales, and which predictions remain level-specific. ELDWConv, RSCB, SA-LSCD, and C3k2-DAPE are names for the resulting module configurations, not claims that depthwise convolution, ECA, CBAM, residual bottlenecks, or softmax branch selection were invented here. The novelty claim therefore concerns the coordinated interfaces and their measured accuracy–complexity behavior rather than new primitive operators. The main contributions are as follows.

(1) LESCNet combines ELDWConv and RSCB as a lightweight backbone configuration. ELDWConv uses the established depthwise–pointwise factorization and ECA recalibration [21,22,23], while RSCB uses a 5 × 5 depthwise convolution, two 1 × 1 projections, and a residual path. These choices allocate low-cost local context and channel interaction to high-resolution feature extraction; their individual operators are established rather than new primitives.
(2) SA-LSCD combines intermediate head sharing with the established CBAM module [24]. The main transformation is reused across P3, P4, and P5, while final classification and regression predictors remain scale-specific. We use CBAM without changing its attention formulation. The design choice is the boundary between shared processing and level-specific prediction.
(3) C3k2-DAPE places a four-branch, softmax-weighted transformation in selected neck fusion bottlenecks, followed by ECA channel recalibration and a residual path. This follows the general principle of input-conditioned selection in selective-kernel and dynamic-convolution methods [25,26]. Its role in SLA-YOLO is to concentrate this processing at fusion sites while the head focuses on parameter reuse; the ablations evaluate the complete neck configuration, not a newly invented branch-selection rule.
(4) Fixed-split experiments on SSDD and HRSID evaluate all eight combinations of LESCNet, SA-LSCD, and C3k2-DAPE in a complete 2^3^ factorial ablation. The analysis reports standalone and pairwise configurations, conditional removals from the complete model, and descriptive main and interaction effects. A three-seed experiment separately repeats the four progressive configurations on newly randomized 8:1:1 partitions, and reports means, sample standard deviations, and paired stage differences. Size-stratified and inshore/offshore comparisons directly evaluate small-target and coastal-background performance against YOLO11n. Four no-fine-tuning transfer settings compare source-trained SLA-YOLO with YOLO11n on HRSID, SSDD, LS-SSDD-v1.0, and SAR-Ship-Dataset targets. These measurements describe performance on the specified benchmarks; because the complete factorial cells were each run once, their interaction contrasts do not establish statistical synergy. Scene-dependent branch specialization and transfer to unseen sensors also remain to be demonstrated in isolation.

## 2. Related Work

### 2.1. Lightweight SAR Ship Detection

Lightweight detection first raises a representation trade-off. MobileNet and Xception use depthwise-separable convolution to reduce spatial-filtering cost while relying on pointwise operations for channel mixing [21,22]. ShuffleNet V2 further shows that memory access and network fragmentation affect practical speed [10]. ECA and CBAM recalibrate channels or channel-spatial responses, whereas selective-kernel networks and dynamic convolution select transformations from the input [23,24,25,26]. These are established operations; the question for weak SAR ship returns is how to combine low-cost spatial context and channel interaction without letting either stage dominate the computational budget.

The SAR detectors illustrate different answers to this trade-off. YOLOv5-MNE adds ECA and coordinate attention to a MobileNet-derived block [12], and LMSD-YOLO uses depthwise-separable DBA units and an S-MobileNet backbone [13]. GSConv combines standard and depthwise operations [27]; Lite-YOLOv5 uses an L-CSP backbone with pruning and attention [28]; and SSGY combines multiscale-kernel extraction with ECA [29]. These approaches demonstrate that inexpensive operators can retain useful representations, so the concern is not an absence of channel interaction in prior work. Rather, the placement of spatial context modeling and channel refinement differs. LESCNet places pointwise mixing and ECA inside ELDWConv, then uses a residual 5 × 5 depthwise-context block (RSCB) to preserve local scattering structure at low cost. This pairing is the specific response to the risk that aggressive factorization weakens weak-target cues.

A second trade-off concerns where fusion adapts to image content. FPN and PANet use prescribed top-down and bottom-up routes [7,8], while BiFPN learns fusion coefficients that are fixed after training [30]. Among SAR detectors, SSGY makes neck processing lighter through GSConv [29]; LMSD-YOLO’s DSASFF adaptively weights scale features before prediction [13]; and AC-YOLO uses channel-aware dynamic processing in its CCFM neck [19]. Adaptive SAR fusion therefore already exists. C3k2-DAPE addresses a narrower choice: at selected neck fusion bottlenecks, four lightweight transformations compete through input-dependent softmax weights before ECA refinement and residual integration. Unlike scale-level reweighting alone, this places adaptation within the feature transformation that follows cross-scale aggregation, while limiting the extra branches to a few fusion locations.

The third trade-off lies in the detection head. The independent decoupled heads of YOLOv8n repeat convolutional processing at three scales, as discussed by LH-YOLO [17]. LH-YOLO’s RSCD addresses this cost by sharing convolutional weights while retaining separate batch normalization; its reported results show that sharing can be effective. Sharing all the way through the final class and box predictors would, however, impose the same terminal mapping on P3, P4, and P5 despite their different feature distributions. This is a design risk, not a reported failure of RSCD. SA-LSCD therefore reuses an intermediate transformation, applies CBAM recalibration [24], and preserves scale-specific final predictors. AC-YOLO [19] is the nearest YOLO11-based SAR comparator: its ACmix replaces C2PSA, CCFM performs cross-scale alignment and channel-aware dynamic processing, and MPDIoU changes the regression loss. SLA-YOLO differs through LESCNet’s lightweight spatial-channel backbone encoding, four-way input-dependent selection inside selected C3k2 neck bottlenecks, and partial sharing of the prediction head. The AC-YOLO study does not report an analysis of that head-sharing boundary; Section 4.5 evaluates the reimplemented AC-YOLO and SLA-YOLO under the same experimental configuration.

### 2.2. Adaptive Fusion and Shared Prediction

FPN, PANet, and BiFPN aggregate features across resolutions through top-down, bottom-up, or weighted bidirectional routes [7,8,30]. Nevertheless, most internal convolutions remain fixed after training. Selective-kernel networks and dynamic convolution use input-conditioned weights to choose among receptive fields or convolution kernels [25,26]. C3k2-DAPE applies this established idea only at selected neck bottlenecks and then uses ECA to recalibrate the resulting channels. By construction, its softmax weights scale whole branch outputs and ECA scales channels after aggregation. This architectural distinction does not show that the learned weights encode sparse and dense scenes differently; that interpretation requires direct weight analysis.

Detection-head design similarly balances parameter reuse with scale specialization. YOLOv8n uses separate prediction branches for its three feature levels, whereas LH-YOLO’s RSCD head shares convolutional weights but retains separate batch-normalization layers [17]. SA-LSCD shares the intermediate transformation across P3, P4, and P5, but keeps the final classification and regression predictors independent; it does not assume that one terminal predictor should map all three feature distributions identically. CBAM provides established channel-spatial recalibration after the shared transformation [24]. The design distinction is the chosen sharing boundary together with adaptive branch selection in the neck, rather than a new attention or sharing operation.

Recent SAR-specific studies also emphasize small ships and oriented localization. Guan et al. proposed an enhanced YOLO network for small-ship representation [31] and an oriented detector based on edge deformable convolution and point-set representation [32]. Both methods are included in the comparative evaluation in Section 4.5 as literature baselines. Superscript P identifies numerical values taken directly from the original papers: the HRSID HBB results of Enhanced YOLO are from [31], and the SSDD OBB results of the oriented detector are from [32]. Because the original dataset partitions, image settings, training schedules, hardware, and box geometries differ from the present study, these values provide contextual comparisons rather than controlled rankings.

### 2.3. Methodological Positioning Relative to Existing Designs

SLA-YOLO is positioned as a system-level redesign of YOLO11n rather than a collection of newly invented operators. Its essential distinction is the simultaneous choice of three interfaces: low-cost spatial–channel encoding before feature-pyramid fusion, input-conditioned transformation selection inside selected neck bottlenecks after cross-scale aggregation, and partial sharing before scale-specific terminal prediction. These choices determine where computation is spent and which information is shared; the attention and convolution primitives themselves remain established.

Compared with lightweight YOLO variants that primarily replace expensive convolutions or compress the backbone and neck, including YOLOv5-MNE, LMSD-YOLO, Lite-YOLOv5, and SSGY [12,13,28,29], SLA-YOLO couples backbone reduction with explicit head sharing and localized adaptive neck processing. LESCNet uses depthwise–pointwise mixing and ECA inside ELDWConv, followed by residual 5 × 5 depthwise refinement, so local context and channel recalibration are completed before the features enter the pyramid. The difference is this ordered backbone configuration and its role in the complete detector, not a new form of depthwise convolution or ECA.

Compared with shared detection heads, particularly the RSCD head in LH-YOLO [17], SA-LSCD chooses a different sharing boundary. Per-level alignment is followed by one shared intermediate transformation and unchanged CBAM recalibration, while the final classification and regression predictors remain independent for P3, P4, and P5. This preserves level-specific terminal mappings rather than sharing the complete prediction path. Thus, the contribution lies in partial sharing with scale-specific outputs and its coordination with adaptive neck processing, not in proposing CBAM or convolutional weight sharing.

Compared with selective-kernel and dynamic-convolution methods [25,26], C3k2-DAPE does not propose a new softmax gating rule. It applies input-dependent weights to four complete transformation branches at selected post-fusion bottlenecks; ECA then recalibrates individual channels after branch concatenation, and a residual path preserves an ungated route. Branch weights therefore select among transformation paths, whereas ECA acts on channels after aggregation. Compared with FPN, PANet, BiFPN, DSASFF, and CCFM [7,8,13,19,30], the adaptive operation is placed inside the transformation following cross-scale aggregation rather than being limited to fusion routes or direct weighting of feature levels.

The coordination claim refers to these non-overlapping assignments: LESCNet reduces high-resolution feature-extraction cost, C3k2-DAPE concentrates input-dependent branch selection at a small number of neck fusion sites, and SA-LSCD removes repeated intermediate head processing while retaining scale-specific outputs. The complete 2^3^ factorial experiments in Section 4.4.4 and Section 4.4.5 evaluate every standalone, pairwise, and three-component configuration on both fixed splits. The full model has the highest mAP point estimates, and all three average main effects are positive. The interaction pattern is non-additive rather than uniformly synergistic, and one run per factorial cell does not establish statistical significance or primitive-level novelty.

## 3. Methodology

### 3.1. Overall Architecture of SLA-YOLO

**Figure 1:**
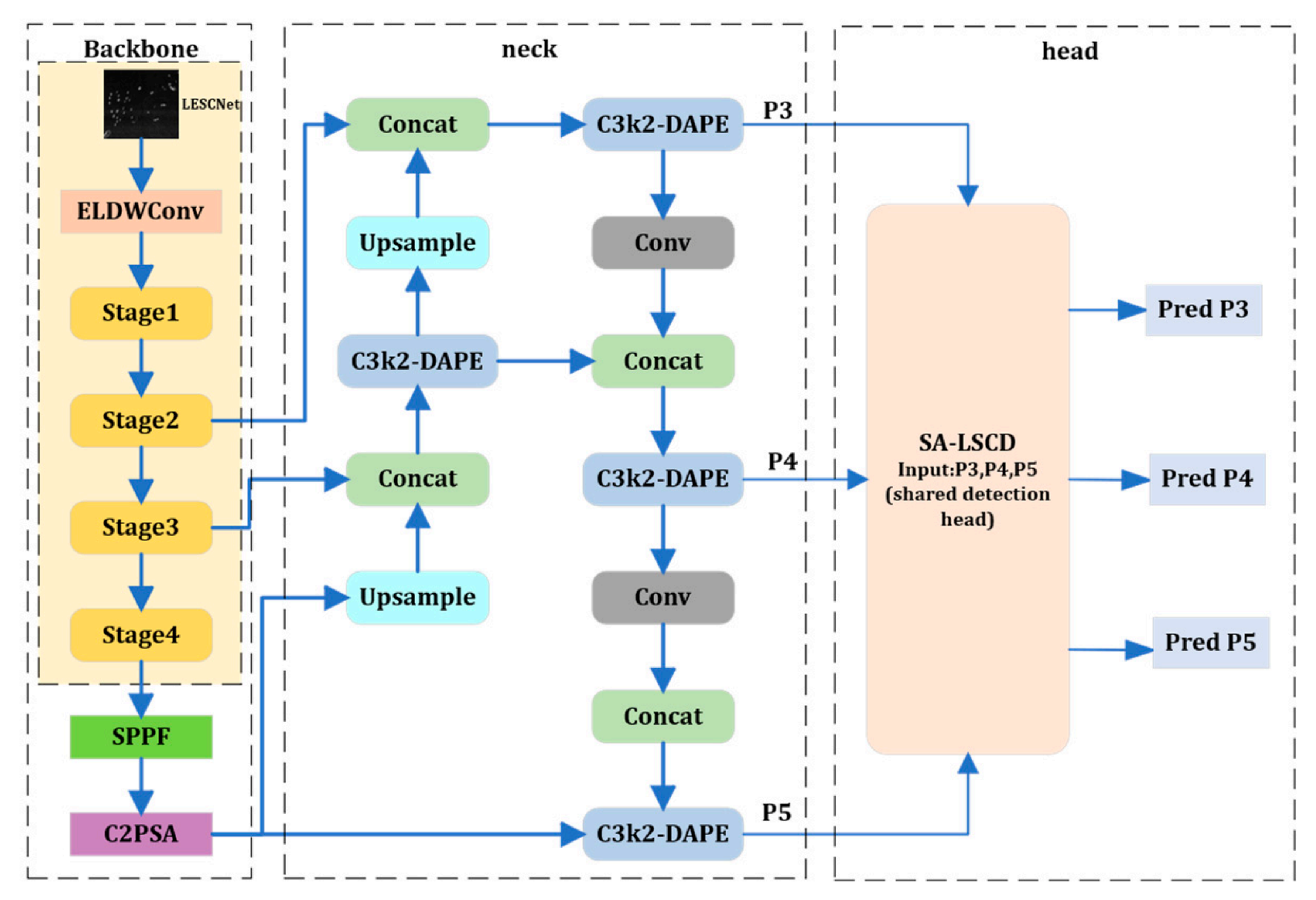
Network architecture of SLA-YOLO.

SLA-YOLO retains the one-stage prediction pipeline of YOLO11n [33] but replaces the original backbone, selected neck blocks, and detection head with LESCNet, C3k2-DAPE, and SA-LSCD, respectively. Figure 1 presents the overall architecture. Hereafter, LESCNet denotes the complete lightweight backbone, while ELDWConv and RSCB denote its constituent blocks. An input SAR image is processed by the LESCNet stem and four hierarchical stages. Progressive downsampling increases the receptive field and channel capacity while generating multilevel features. The neck combines top-down semantic transfer with bottom-up localization reinforcement and produces P3, P4, and P5. For a 640 × 640 input, these levels have strides of 8, 16, and 32. SA-LSCD applies a shared intermediate transformation and then performs scale-specific classification and regression. Section 3.2, Section 3.3 and Section 3.4 separately describe the LESCNet backbone, SA-LSCD detection head, and C3k2-DAPE neck; feature-pyramid fusion and C3k2-DAPE are discussed only in the neck subsection.

The three components are allocated to different stages with explicit interface constraints. LESCNet reduces arithmetic cost during high-resolution feature extraction and completes local spatial-channel encoding before pyramid fusion. C3k2-DAPE introduces input-conditioned branch selection only at selected neck blocks after feature scales have been combined, avoiding repeated dynamic processing throughout the backbone and head. SA-LSCD shares only the intermediate head transformation and retains separate final classification and regression predictors for P3, P4, and P5. The coordination claim refers to this placement and sharing boundary: each component addresses a different source of redundancy while preserving an ungated residual route and scale-specific terminal mappings. The complete 2^3^ factorial ablation in Section 4.4 evaluates all eight combinations. Each component has a positive average main effect on both mAP measures and datasets, while the negative pairwise and positive three-way interaction contrasts show that the modules’ returns depend on which other modules are present. The full model nevertheless has the highest mAP point estimates on both fixed splits. Repeated runs for all eight cells would be required for statistical interaction or synergy claims.

Throughout the manuscript, Params and GFLOPs refer to the complete end-to-end detector configuration shown in the corresponding row rather than to the named module in isolation. The YOLO11n baseline contains 2.58 million parameters and requires 6.3 GFLOPs. Replacing only the backbone with LESCNet while retaining the baseline neck and head gives the LESCNet-only configuration with 1.86 million parameters and 4.3 GFLOPs. Replacing the head with SA-LSCD gives LESCNet + SA-LSCD with 1.70 million parameters and 3.6 GFLOPs. The neck-stage ablation in Section 4.4.3 then compares two alternative neck blocks under that fixed backbone-head combination: C3k2-AP gives 1.61 million parameters and 3.6 GFLOPs, whereas the selected C3k2-DAPE configuration gives the complete SLA-YOLO model with 1.70 million parameters and 3.6 GFLOPs. These values therefore identify different end-to-end ablation configurations.

SLA-YOLO is a horizontal object detector and directly predicts axis-aligned boxes. A prediction-level geometry-matched comparison with [32] would require decoding each rotated prediction into four vertices ($x_{k}$, $y_{k}$), *k* = 1, …, 4, replacing it with the tight enclosing HBB [$\underset{k}{\min}\;x_{k}$, $\underset{k}{\min}\;y_{k}$, $\underset{k}{\max}\;x_{k}$, $\underset{k}{\max}\;y_{k}$], preserving its class and confidence score, and then applying the same HBB NMS, horizontal ground-truth matching, IoU thresholds, and AP implementation used for SLA-YOLO. However, Ref. [32] reports only aggregate native-OBB precision, recall, and AP50, not the prediction files required for this conversion. The published OBB values are therefore retained with a separate geometry marker and are not treated as converted HBB results or included in the direct HBB ranking.

### 3.2. LESCNet Lightweight Backbone

Backbone design is critical because small SAR ships may occupy only a few pixels, and their scattering signatures can be damaged by excessive compression. Standard convolution jointly models spatial and channel dimensions, whereas depthwise-separable convolution decomposes this operation into channel-wise spatial filtering and 1 × 1 channel mixing [21,22]. Although this decomposition reduces arithmetic cost, depthwise filtering alone cannot exchange information across channels, and a single pointwise projection can treat all output channels equally. LESCNet therefore combines efficient spatial filtering, explicit channel recalibration, large-kernel local context, and residual refinement.

**Figure 2:**
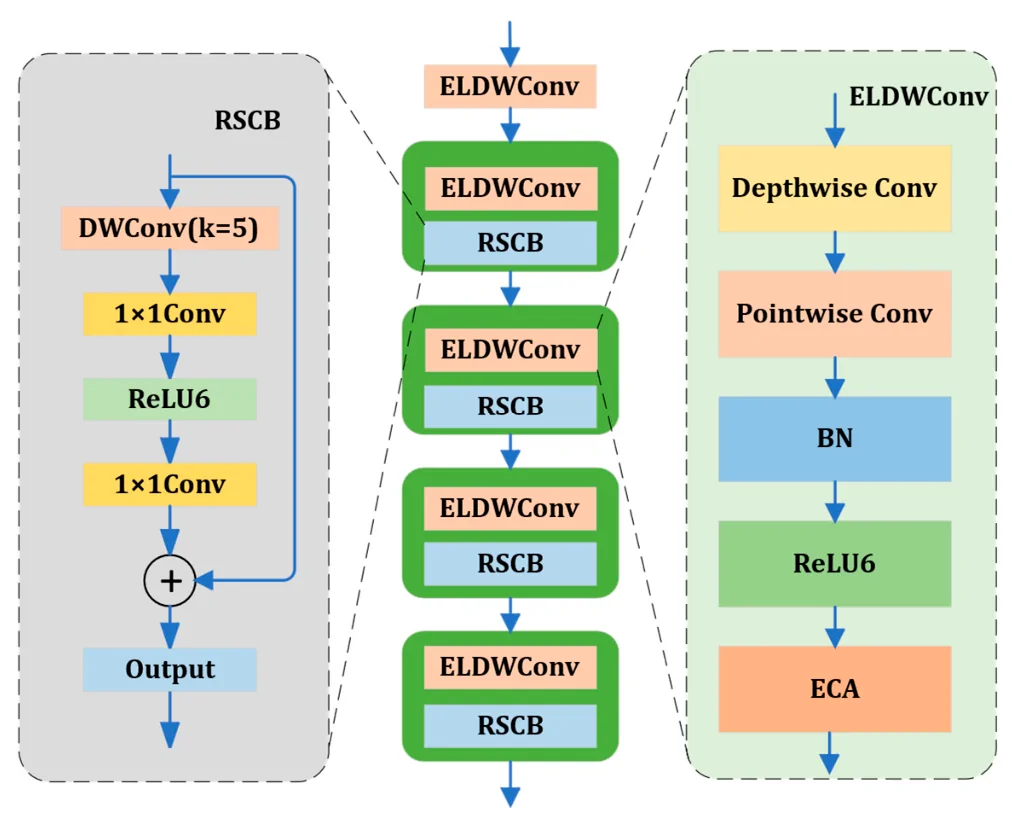
Structure of LESCNet.

As shown in Figure 2, LESCNet contains a compact stem followed by four stages. Each stage begins with ELDWConv for downsampling and spatial-channel encoding, followed by one or more RSCB units for residual refinement. Classification-specific global pooling and fully connected layers are removed. The multi-level outputs are delivered directly to the neck.

ELDWConv combines depthwise–pointwise convolution with established ECA channel recalibration [23]. Given an input feature map, its lightweight spatial-channel transformation is expressed as

(1) $$F_{d}=DWConv_{k_{d}\times k_{d}}^{\left(s\right)}(X),$$

(2) $$F_{p}=ReLU6[BN(PWConv(F_{d}))],$$

where *k*_*d*_ and *s* denote the kernel size and stride, respectively.

**Figure 3:**
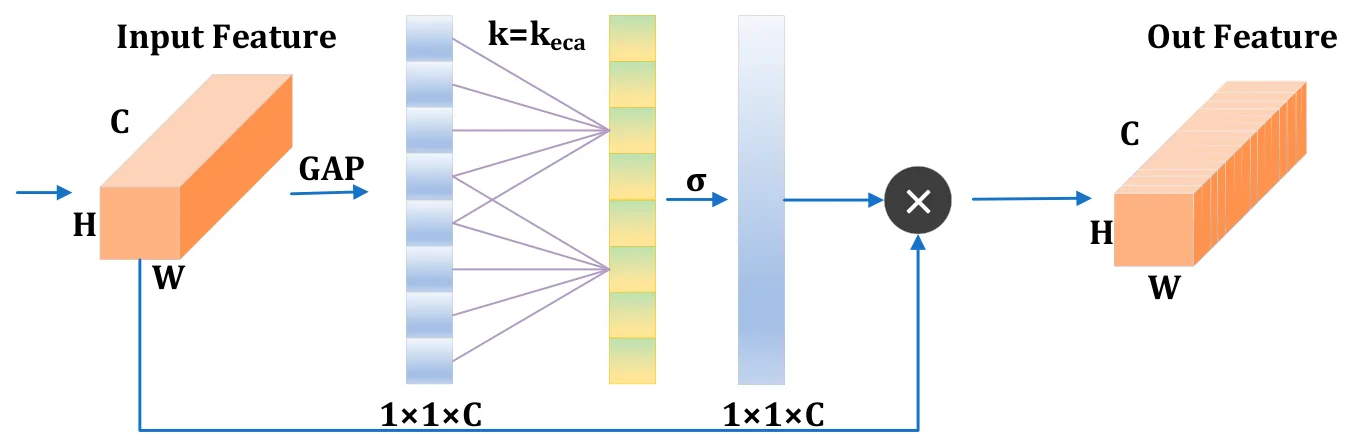
Structure of the ECA module.

Depthwise convolution extracts spatial information independently within each channel, while pointwise convolution enables cross-channel interaction. Because the projected channels would otherwise be treated equally, ECA is applied after pointwise projection. As illustrated in Figure 3, global average pooling first compresses spatial information into a channel descriptor. A lightweight one-dimensional convolution then models local cross-channel dependencies, and a sigmoid function generates channel weights:

(3) $$a=\sigma [Conv1D_{k_{eca}}(GAP(F_{p}))],Y_{E}=F_{p}⊙a,$$

where σ(⋅) denotes the sigmoid function, ⊙ represents channel-wise weighting, and $k_{eca}$ is adaptively determined as

(4) $$k_{eca}={\left|\frac{log_{2}C_{out}+b}{\gamma }\right|}_{odd}.$$

ECA captures local cross-channel dependencies without dimensionality reduction [23]. ELDWConv uses the established depthwise–pointwise sequence [21,22] with ECA recalibration [23]. Its role in LESCNet is the selected placement and combination of these operations, not a new convolution or attention mechanism.

Following ELDWConv, RSCB is employed to further model spatial context and channel dependencies. For an input $X\in R^{C\times H\times W}$, a 5×5 depthwise convolution first enlarges the local receptive field with limited computational overhead:

(5) $$F_{s}=DWConv_{5\times 5}(X).$$

Two 1×1 pointwise convolutions are then used to compensate for the absence of cross-channel interaction in depthwise convolution:

(6) $$F_{c}=PWConv_{2}[ReLU6(PWConv_{1}(F_{s}))].$$

Since the input and output dimensions remain unchanged, an identity shortcut is introduced as

(7) $$Y_{R}=X+F_{c}.$$

The depthwise convolution captures local spatial structure, the pointwise projections perform nonlinear channel interaction, and the residual connection preserves the input while providing a direct gradient path. RSCB resembles an inverted-residual-style bottleneck in its use of a depthwise filter, pointwise projections, and a skip path; the factorized convolution operations themselves are established [21,22]. Its 5 × 5 local-context setting and position after ELDWConv are the design choices evaluated here, rather than a fundamentally new residual principle.

Assuming bias-free convolution, the complete parameter quantities of ELDWConv and RSCB are

(8) $$P_{ELDWConv}=k_{d}^{2}C_{in}+C_{in}C_{out}+2C_{out}+k_{eca},$$

(9) $$P_{RSCB}=25C+2CC_{h},$$

where $C_{h}$ denotes the intermediate channel dimension in RSCB. The terms in (8) correspond to depthwise convolution, pointwise convolution, batch normalization, and ECA, respectively, whereas the residual connection and activation functions introduce no trainable parameters.

If the i-th stage contains one ELDWConv module and $n_{i}$ repeated RSCB modules, its parameter count is expressed as

(10) $$P_{stage,i}=P_{ELDWConv,i}+n_{i}P_{RSCB,i}.$$

Therefore, the total parameter count of the LESCNet backbone is

(11) $$P_{LESCNet}=P_{stem}+\sum_{i=1}^{4}\left(P_{ELDWConv,i}+n_{i}P_{RSCB,i}\right).$$

Here, $n_{i}$ denotes the number of repeated RSCB modules in the i-th stage. Using the actual channel widths and repetition numbers, the stem and four stages contain 190, 2771, 7667, 80,995, and 110,533 parameters, respectively, yielding 202,156 parameters in total. The complete SLA-YOLO model contains 1,701,063 trainable parameters according to model profiling.

Through the coordinated use of ELDWConv and RSCB, LESCNet provides efficient spatial–channel representation while retaining hierarchical features required for small and multiscale SAR ship detection.

### 3.3. SA-LSCD Shared Detection Head

**Figure 4:**
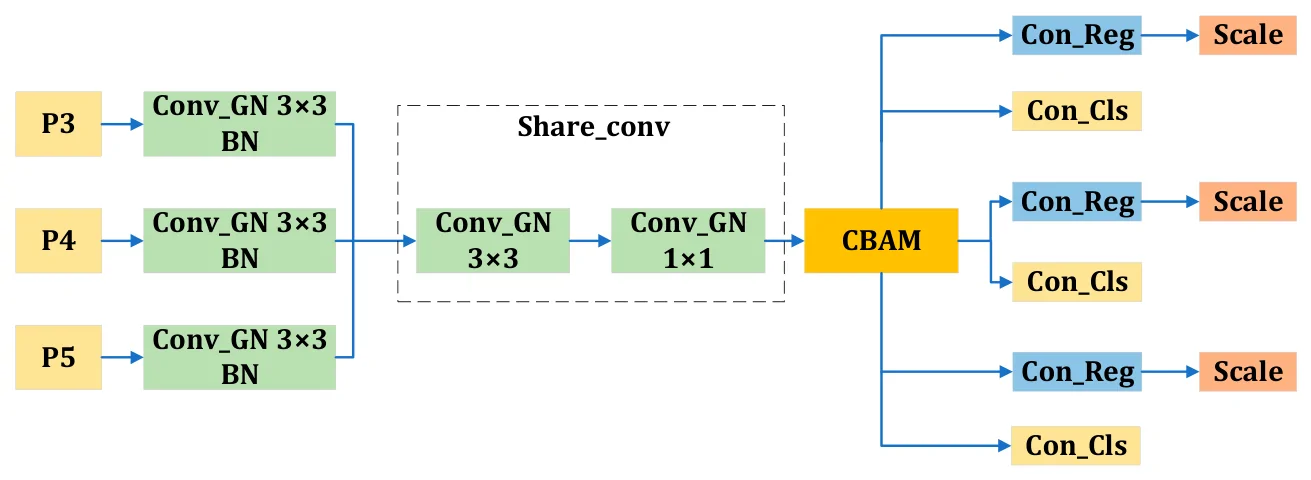
Structure of the SA-LSCD head.

Conventional multiscale detection heads frequently repeat nearly identical convolutional blocks at P3, P4, and P5. Independent branches retain scale-specific learning but duplicate parameters, whereas complete sharing can force features with different resolutions and semantic depth into the same prediction distribution. SA-LSCD therefore adopts partial sharing: an alignment convolution maps each neck output to a common channel width; the main intermediate transformation is shared; and the final classification and regression predictors remain independent for each level. Figure 4 illustrates this structure.

Let $P_{l}$ denote the input at level l∈{3,4,5}. After alignment, the shared feature is

(12) $$S_{l}=\phi_{shared}(\phi_{align,l}(P_{l})),l\in \left\{3,4,5\right\},$$

where $\phi_{shared}$ has the same parameters for all levels. The scale-specific outputs are

(13) $${\hat{Β}}_{l}=\psi_{reg,l}(A_{l}),{\hat{C}}_{l}=\psi_{cls,l}(A_{l}),$$

where $A_{l}$ is the attention-refined feature. This arrangement removes repeated intermediate convolution while preserving separate output calibration for small, medium, and large targets.

**Figure 5:**
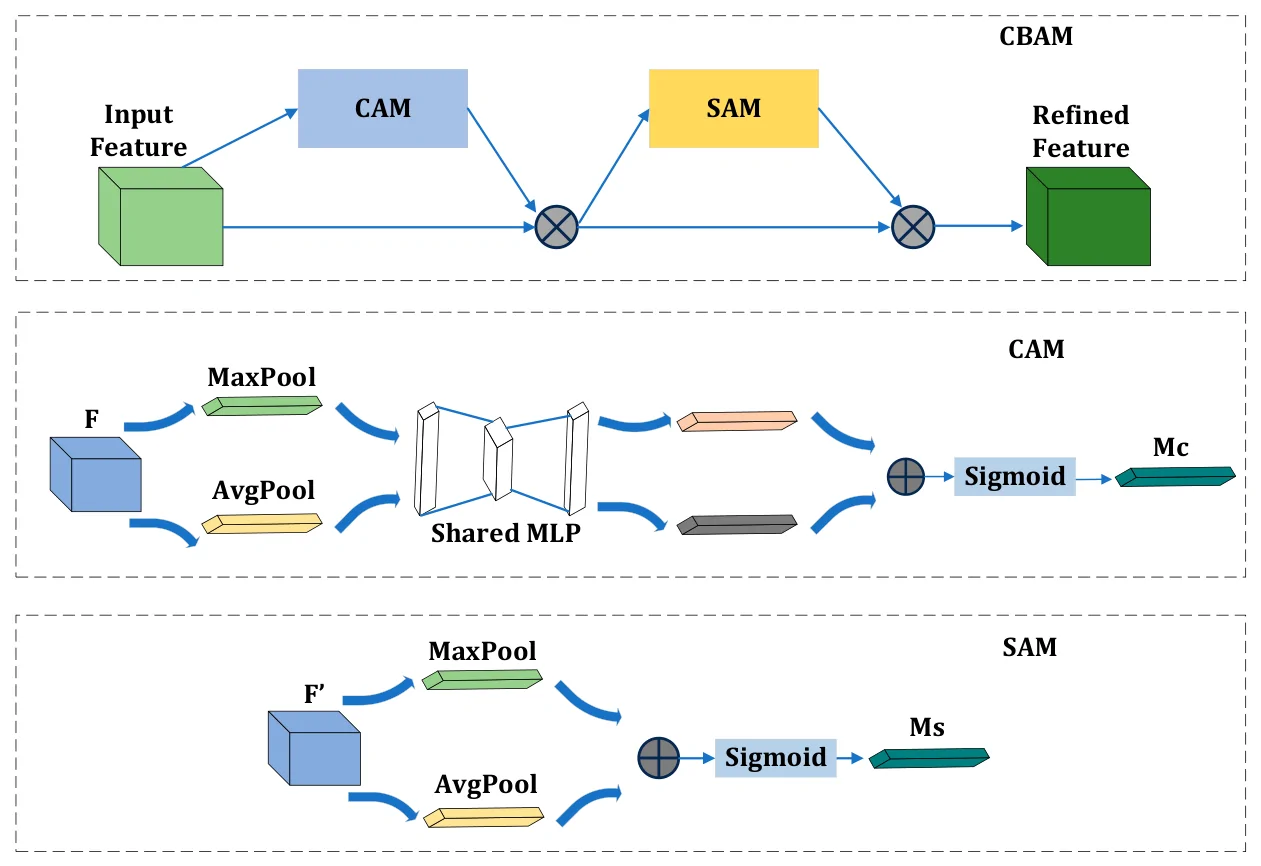
Structure of the CBAM module.

SAR scenes often contain strong but irrelevant responses from coastlines, buildings, oil platforms, waves, and azimuth ambiguities. SA-LSCD uses the standard sequential channel and spatial attention mechanism of CBAM [24], shown in Figure 5, after the shared intermediate transformation. CBAM’s pooling, channel attention, spatial attention, and ordering are unchanged. The architectural choice being evaluated is where this existing module is placed relative to shared processing and scale-specific predictors.

For an input $F\in R^{C\times H\times W}$, channel attention is computed from global average and maximum pooling:

(14) $$M_{c}\left(F\right)=\sigma \left(MLP\left(AvgPool\left(F\right)\right)+MLP\left(MaxPool\left(F\right)\right)\right).$$

The channel-refined feature is

(15) $$F_{c}=M_{c}(F)⊙F.$$

Spatial attention then aggregates information along the channel dimension and uses a 7 × 7 convolution:

(16) $$M_{s}(F_{c})=\sigma (f^{7\times 7}([AvgPool_{c}(F_{c});MaxPool_{c}(F_{c})])).$$

(17) $$F_{o}=M_{s}(F_{c})⊙F_{c}.$$

The channel-first ordering follows the original CBAM design [24]. Placing the module after the shared convolution gives it consistently sized inputs across P3, P4, and P5, while independent final predictors retain level-specific output mappings. Whether this attention placement has a distinct effect beyond parameter sharing is not isolated by the current head ablation.

### 3.4. C3k2-DAPE Adaptive Neck

Feature-pyramid fusion is essential for SAR ship detection because scenes may contain small offshore vessels, medium cargo ships, and large densely berthed targets. FPN propagates high-level semantics downward, PANet adds a bottom-up localization path, and BiFPN introduces weighted bidirectional fusion [7,8,30]. The transformations within these routes are typically fixed after training. Selective-kernel networks and dynamic convolution already demonstrate input-conditioned weighting of alternative receptive fields or kernels [25,26]; C3k2-DAPE adapts that general strategy to selected YOLO11n neck fusion blocks.

**Figure 6:**
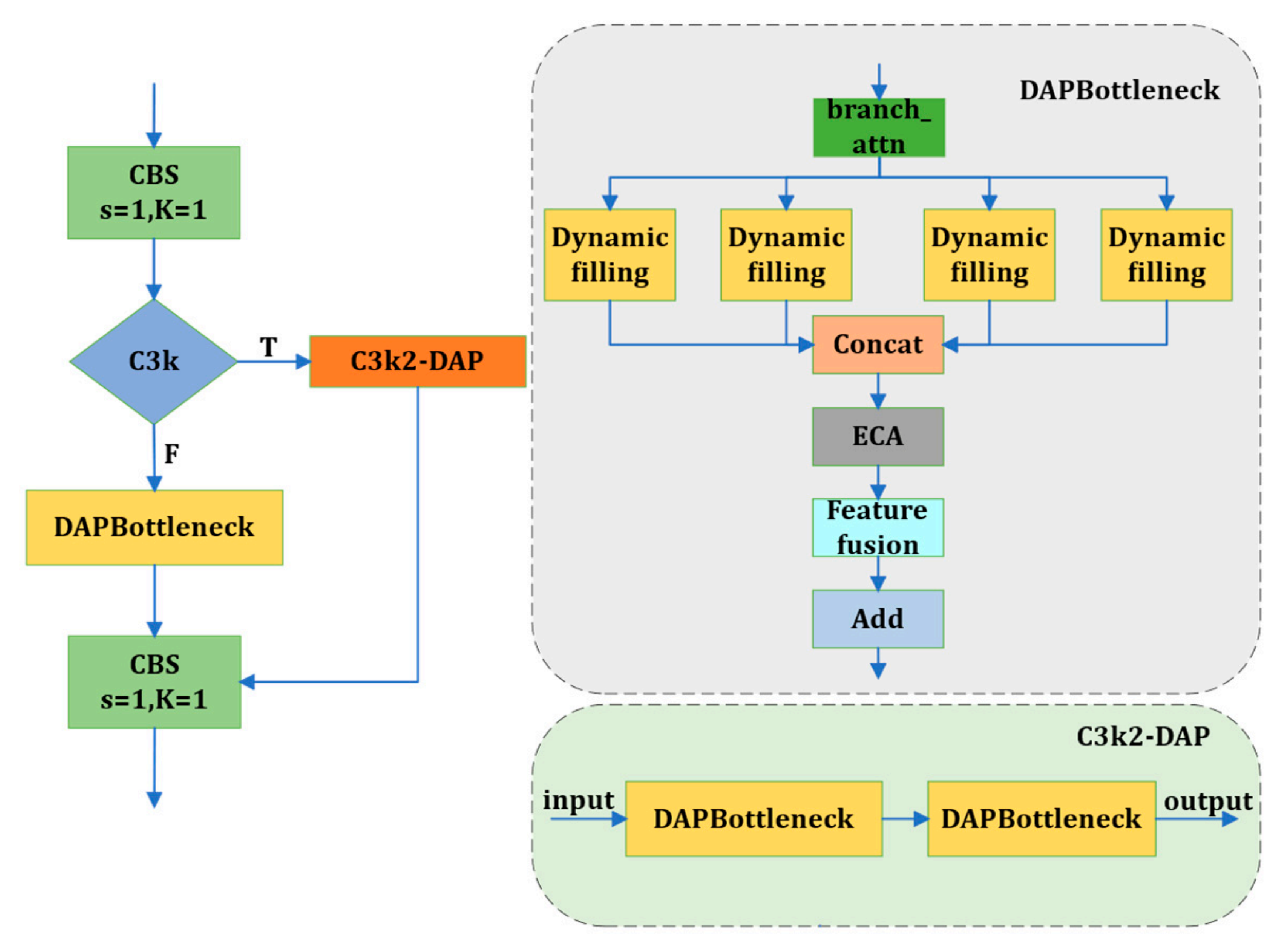
Structure of C3k2-DAPE. T and F denote the true and false routes of the C3k selector, respectively.

C3k2-DAPE introduces this principle inside the compact neck. As shown in Figure 6, an input with $C_{in}$ channels is first projected to $2C_{h}$ channels, where

(18) $$C_{h}=\left\lfloor eC_{out}\right\rfloor .$$

Here, e is the expansion ratio, and $C_{h}$ is the hidden channel width. The projected feature is then split, and a dynamic attention-guided parallel bottleneck produces four differently parameterized branch outputs. These branches offer alternative transformations; the current experiments do not establish that each learns a distinct semantic or scene-specific role. Let

(19) $$B_{i}=\phi_{i}(X),i=1,2,3,4.$$

A branch-attention unit applies a 1 × 1 convolution and global average pooling to obtain an input-conditioned score $z_{i}$. softmax normalization produces

(20) $$\alpha_{i}=\frac{\exp(z_{i})}{\sum_{j=1}^{4}\exp(z_{j})},\sum_{i=1}^{4}\alpha_{i}=1.$$

The weighted branches are concatenated:

(21) $$B=Concat(\alpha_{1}B_{1},\alpha_{2}B_{2},\alpha_{3}B_{3},\alpha_{4}B_{4}).$$

ECA then recalibrates the concatenated channels, and a projection fuses them. If the dimensions are compatible, the original input is added:

(22) Y=X+Proj(ECA(B)).

By construction, branch weights scale whole transformation paths before concatenation, whereas ECA recalibrates individual channels after aggregation. The residual connection retains an unweighted route for the original input. These are different mathematical operations, but the present experiments do not reveal what each learns. We have not measured the four learned softmax weights for sparse versus dense scenes and therefore do not claim that a particular branch specializes in either scene type. A direct test would record weights for each C3k2-DAPE block on held-out images, group images by annotated ship count and offshore/inshore context, and compare branch-weight distributions and entropy while showing representative images.

The feature-processing mode is controlled by the C3k flag. When C3k = False, a DAPBottleneck performs the four-branch adaptive transformation. When C3k = True, the C3k2-DAPE route stacks two DAPBottleneck units for deeper extraction. The aggregated feature is finally projected to the required output channels by a 1 × 1 convolution.

The additional branches increase memory access and operation scheduling. Therefore, the relationship between C3k2-DAPE, FLOPs, and FPS must be established by measurement rather than inferred from arithmetic complexity. FLOPs describe arithmetic workload, whereas latency also depends on kernel efficiency, parallelism, memory bandwidth, tensor layout, and framework implementation. All reported FPS values use the common synchronized network-only protocol specified in Section 4.3. The mechanism analysis does not attribute the accuracy gain to a verified scene-dependent branch preference.

### 3.5. Detection Objective

SLA-YOLO retains the baseline YOLO11 detection objective so that the architectural comparison is not confounded by a new loss function. The total objective is written as

(23) $$L=\lambda_{box}L_{Iou}+\lambda_{cls}L_{BCE}+\lambda_{dfl}L_{DFL},$$

where $L_{IoU}$ optimizes bounding-box overlap, $L_{BCE}$ performs binary classification supervision, and $L_{DFL}$ is distribution focal loss for box-coordinate estimation. The weighting coefficients follow the baseline configuration used for all compared architectural variants. Retaining the same objective isolates the contribution of LESCNet, SA-LSCD, and C3k2-DAPE.

## 4. Results and Analysis

### 4.1. Experimental Environment

Experiments were implemented with the Ultralytics detection framework and PyTorch 2.5 under Ubuntu 22.04, Python 3.12, and CUDA 12.4. Training and evaluation were performed on an NVIDIA RTX 3090 GPU with 24 GB of memory. Results marked R or S in the method-comparison analysis were generated in this study. These models were trained for 400 epochs using SGD with an initial learning rate of 0.001 and a training batch size of 4; they share the fixed data split, 640 × 640 input, preprocessing, checkpoint rule, training configuration, and evaluation code. Results marked P were taken directly from the original papers [31,32] and retain their source-specific protocols. Throughput for the R- and S-marked rows was measured at inference batch size 1 and 640 × 640 input in FP32, using 100 warm-up forward passes followed by 500 CUDA-synchronized timed forward passes. The timing covers the network forward pass only and excludes input preprocessing, host-to-device data transfer, and NMS postprocessing. The published FPS marked P is reported as given in [31] and is not directly comparable with the RTX 3090 measurements. The ablation, comparison, and transfer experiments report single-run values, with the P-marked literature rows retaining their original protocols; Section 4.7 reports means and sample standard deviations across seeds 0, 1, and 2 for the four principal configurations on SSDD and HRSID. Paired stage differences were computed within each seed before aggregation.

### 4.2. Experimental Datasets

The within-dataset experiments used two publicly available SAR ship datasets. SSDD contains 1160 images and 2456 ship instances from RadarSat-2, TerraSAR-X, and Sentinel-1 imagery [3]. It includes offshore and inshore scenes, multiple polarizations, and spatial resolutions of approximately 1–15 m. HRSID contains 5604 image patches and 16,951 ship instances from Sentinel-1B, TerraSAR-X, and TanDEM-X imagery [4], including offshore, nearshore, and dense-ship scenes. The datasets overlap in the Sentinel-1 and TerraSAR-X sensor families. Horizontal bounding boxes were used for the reported SLA-YOLO results. Section 4.6 additionally evaluates no-fine-tuning transfer between SSDD and HRSID and to LS-SSDD-v1.0 and SAR-Ship-Dataset, and it summarizes the published charac-teristics of the two additional target benchmarks.

For the study-generated single-run SSDD and HRSID results, each dataset was divided at the image level into one fixed 8:1:1 training, validation, and test partition reused for every R- and S-marked model. SSDD therefore has approximately 116 test images. The exact image identifiers for these fixed partitions were stored as train, validation, and test lists so that the same samples can be reconstructed without relying on an unspecified random-number-generator state. For the three-seed experiment described in Section 4.7, seeds 0, 1, and 2 each generated a new 8:1:1 partition, and all four configurations shared that seed’s train, validation, and test images. The fixed and seed-specific split manifests, together with the corresponding configuration files, will be released with the final manuscript. Images were resized to 640 × 640 pixels, and stochastic augmentation was applied only to training images. The P-marked literature rows are exceptions: [31] reports HRSID results using its original 65:35 training-test division and 800 × 800 image chips, while [32] reports SSDD results using an 8:2 training-test division, 36 training epochs, and native oriented-box evaluation. These published values were not recomputed under the present split. Because HRSID contains patches from larger acquisitions, image-level partitioning alone does not establish source-scene independence; acquisition provenance should also be audited.

### 4.3. Evaluation Metrics

Precision (P) measures the proportion of predicted positives that are correct and therefore reflects resistance to false alarms caused by background clutter. It is defined in Equation (24):

(24) $$P=\frac{TP}{TP+FP}$$

where TP is the number of correctly detected targets, and FP is the number of background regions incorrectly predicted as ships. Higher precision indicates fewer false alarms.

Recall (R) measures the proportion of ground-truth targets that are detected and is particularly relevant to missed small or densely distributed ships. It is defined in Equation (25):

(25) $$R=\frac{TP}{TP+FN}$$

where FN is the number of ground-truth targets that remain undetected. Higher recall indicates fewer missed detections.

Average Precision (AP) integrates the precision–recall curve for one class, while mean Average Precision (mAP) averages AP across classes. Because both datasets contain a single ship category, the reported mAP corresponds numerically to ship AP under the selected IoU protocol. mAP@0.5 is calculated at IoU = 0.50, whereas mAP@0.5:0.95 averages AP over IoU thresholds from 0.50 to 0.95 in increments of 0.05. Equations (26) and (27) define AP and mAP:

(26) $$AP=\int_{0}^{1}P(R)dR$$

(27) $$mAP=\frac{1}{N}\sum_{i=1}^{N}AP_{i}$$

Efficiency is evaluated using the number of trainable parameters (Params), floating-point operations at 640 × 640 input resolution (GFLOPs), and measured throughput in frames per second (FPS). For every study-generated FPS entry marked R or S, inference uses batch size 1, 640 × 640 input, and FP32 precision on the RTX 3090. We execute 100 warm-up forward passes, and then time 500 forward passes with CUDA synchronization. The reported network-only throughput excludes input preprocessing, host-to-device transfer, and NMS postprocessing; FPS is derived from the synchronized per-image forward-pass time. Params and GFLOPs describe model size and arithmetic workload, whereas these FPS values characterize this specific desktop-GPU implementation and should not be extrapolated directly to embedded hardware.

Precision, recall, mAP@0.5, mAP@0.5:0.95, Params, GFLOPs, and FPS jointly characterize accuracy, localization, complexity, and measured speed. The ablation analyses report accuracy and complexity, while the method-comparison analysis reports FPS measured under the common protocol. Under this protocol, YOLO11n achieves 42.9 FPS on SSDD and 43.8 FPS on HRSID, whereas SLA-YOLO achieves 66.8 and 63.4 FPS, respectively. The P-marked Enhanced YOLO FPS is copied from [31] and retains that paper’s hardware and timing conditions, so it is not used for a direct throughput ranking. The ablation and transfer analyses contain study-generated single-run accuracies; in the method-comparison analysis, R- and S-marked rows are study-generated, whereas P-marked rows retain source-reported values. Section 4.7 summarizes three inde-pendently seeded partitions for the principal within-dataset configurations, using sample standard deviation (*n* − 1 denominator) and seed-matched differences. Section 4.8 reports size- and scene-stratified AP/mAP for YOLO11n and SLA-YOLO on the same three seed-specific partitions.

### 4.4. Ablation Results

#### 4.4.1. Lightweight Convolution Variants

Table 1 and Table 2 isolate the backbone-stage comparison while retaining the baseline neck and detection head. The ELDWConv row therefore represents the LESCNet-only detector, not the complete SLA-YOLO model. Replacing the baseline backbone with LESCNet reduces the end-to-end detector from 2.58 to 1.86 million parameters and from 6.3 to 4.3 GFLOPs. After the head is subsequently replaced by SA-LSCD, the LESCNet + SA-LSCD configuration contains 1.70 M parameters and requires 3.6 GFLOPs. Adding the selected C3k2-DAPE neck then forms the complete SLA-YOLO model, whose rounded complexity remains 1.70 M parameters and 3.6 GFLOPs. On SSDD, the LESCNet-only configuration increases mAP@0.5 from 92.9% to 96.8% and mAP@0.5:0.95 from 55.5% to 65.6%. On HRSID, the corresponding metrics increase from 85.6% to 89.1% and from 59.0% to 61.8%.

The ELDWConv configuration shows that arithmetic reduction can coincide with higher detection accuracy in these experiments. It combines depthwise spatial filtering, pointwise channel interaction, and ECA recalibration, but the present comparison does not isolate the contribution of ECA. SSDD mAP@0.5:0.95 rises by 10.1 percentage points and HRSID by 2.8 points relative to the baseline convolution configuration. GSConv has slightly fewer parameters and FLOPs, whereas its HRSID mAP@0.5 is 1.1 points below ELDWConv in the reported run; the stability of this small difference has not been tested. These results support the tested backbone configuration without establishing a new operator-level mechanism.

The measured speed comparison also illustrates that FLOPs and execution time are distinct quantities. Lightweight operators, attention, and multi-branch paths can change GPU utilization, memory traffic, and kernel-launch overhead, so throughput must be interpreted under its stated protocol. The R- and S-marked FPS values in the method-comparison analysis are direct net-work-only measurements on the RTX 3090. The P-marked Enhanced YOLO FPS comes from [31] and is shown only as a source-reported value because its hardware and timing conditions are not identical.

#### 4.4.2. Detection-Head Ablation

Table 3 and Table 4 hold the LESCNet backbone fixed and compare three head settings: the baseline head, an LSCD + ASFF comparison head, and the proposed SA-LSCD head. LSCD + ASFF denotes a lightweight shared-convolution detection head coupled with adaptive spatial feature fusion (ASFF); it is a distinct comparator rather than an alternative name for SA-LSCD. SA-LSCD instead uses partial intermediate sharing, scale-specific final predictors, and the unchanged CBAM attention formulation described in Section 3.3. SA-LSCD reduces the complete detector to 1.70 million parameters and 3.6 GFLOPs and has the highest single-run mAP@0.5:0.95 among the evaluated head variants on both datasets, reaching 65.9% on SSDD and 64.2% on HRSID. Its mAP@0.5 values are 96.5% and 90.6%, respectively.

The head comparison supports a system-level allocation of computation: with LESCNet fixed, SA-LSCD yields higher mAP@0.5:0.95 than the LSCD + ASFF comparator at lower parameter and FLOP counts on both datasets.

#### 4.4.3. Neck Ablation

Table 5 and Table 6 hold LESCNet and SA-LSCD fixed and compare the original neck block with two explicitly named alternatives, C3k2-AP and C3k2-DAPE. Both tables report Params and GFLOPs for every configuration. In Table 6, the original neck, C3k2-AP, and C3k2-DAPE configurations each require 3.6 GFLOPs. C3k2-AP is an alternative neck configuration rather than the final model; it reduces the end-to-end detector to 1.61 million parameters and 3.6 GFLOPs, slightly increases SSDD mAP@0.5, and decreases SSDD mAP@0.5:0.95 from 65.9% to 65.6%. The selected C3k2-DAPE configuration forms SLA-YOLO at 1.70 million parameters and 3.6 GFLOPs, reaching 97.3% mAP@0.5 and 68.2% mAP@0.5:0.95 on SSDD and 90.8% and 64.3% on HRSID. These are the highest single-run mAP@0.5:0.95 point estimates among the evaluated neck variants.

The difference between mAP@0.5 and mAP@0.5:0.95 is informative: a detection may pass IoU 0.50 while a loose or shifted box fails at stricter thresholds. Under the fixed LESCNet and SA-LSCD configuration, adding C3k2-DAPE raises single-run SSDD mAP@0.5:0.95 from 65.9% to 68.2% and HRSID mAP@0.5:0.95 from 64.2% to 64.3%. HRSID recall rises from 80.3% to 82.8%. The HRSID +0.1-point single-run localization contrast differs from the three-seed paired mean of +1.20 ± 0.26 points reported in Section 4.7. The repeated results indicate positive differences in the three tested partitions, but they do not isolate branch weighting, ECA, or scene-specific selection as the causal source of the gain.

Under the common timing protocol, the complete SLA-YOLO model reaches 66.8 FPS for the SSDD-trained checkpoint and 63.4 FPS for the HRSID-trained checkpoint. The corresponding YOLO11n values are 42.9 and 43.8 FPS. Thus, the complete model improves network-only throughput on the tested RTX 3090 while reducing the arithmetic workload from 6.3 to 3.6 GFLOPs. The two checkpoint measurements are reported separately because all timing values are associated with the corresponding controlled dataset experiment. This result supports desktop-GPU efficiency under the stated protocol; it does not substitute for timing on an embedded target.

#### 4.4.4. Complete Factorial Component Analysis

Table 7 and Table 8 report the complete 2^3^ factorial design for three binary factors: LESCNet, SA-LSCD, and C3k2-DAPE. The eight configurations comprise the YOLO11n baseline, each of the three modules alone, all three two-module combinations, and the complete model. Thus, the design includes the progressive path, the two additional standalone controls, and every leave-one-component-out comparison. All configurations within a dataset use the same fixed split, initialization seed, training schedule, checkpoint rule, preprocessing, and evaluation settings, so differences between cells are matched descriptive contrasts.

The standalone results show that no component depends entirely on the other two to improve the baseline. On SSDD, LESCNet, SA-LSCD, and C3k2-DAPE alone obtain mAP@0.5/mAP@0.5:0.95 of 96.8%/65.6%, 95.3%/64.6%, and 95.8%/65.1%, respectively, compared with 92.9%/55.5% for YOLO11n. On HRSID, the corresponding standalone results are 89.1%/61.8%, 89.3%/62.3%, and 88.9%/62.5%, compared with 85.6%/59.0%. The complete model reaches 97.3%/68.2% on SSDD and 90.8%/64.3% on HRSID, the highest mAP point estimates among all eight configurations. Compared with the three two-module models, the complete model improves mAP@0.5:0.95 by 1.4–2.3 points on SSDD and 0.1–0.8 points on HRSID. Section 4.7 separately provides three-seed variability for the four progressive configu-rations; the remaining factorial cells were evaluated once on the fixed split.

#### 4.4.5. Factorial Main Effects and Interactions

The three factors are LESCNet, SA-LSCD, and C3k2-DAPE. For each metric, the main effect of a factor is the difference between its mean response when present and its mean response when absent, averaged over the four settings of the other two factors. Each two-factor interaction is the corresponding difference-in-differences averaged over the third factor, and the three-factor interaction is the change in a two-factor interaction between the two levels of the remaining factor. Parameters and GFLOPs were recomputed for every configuration rather than inferred by subtracting module counts. Because the components replace different backbone, neck, and head operations, their complexity effects are not additive.

All average main effects are positive. For mAP@0.5, the effects of LESCNet, SA-LSCD, and C3k2-DAPE are +1.78, +0.78, and +1.18 percentage points on SSDD and +1.53, +1.93, and +1.23 points on HRSID. For mAP@0.5:0.95, the corresponding effects of LESCNet, SA-LSCD, and C3k2-DAPE are +3.75, +3.00, and +3.70 points on SSDD and +1.53, +1.98, and +1.73 points on HRSID. The LESCNet × SA-LSCD, LESCNet × C3k2-DAPE, and SA-LSCD × C3k2-DAPE pairwise interaction contrasts are negative: −1.35, −1.55, and −0.55 points for SSDD mAP@0.5; −4.30, −3.90, and −3.40 for SSDD mAP@0.5:0.95; −1.15, −1.75, and −1.35 for HRSID mAP@0.5; and −0.75, −1.65, and −1.75 for HRSID mAP@0.5:0.95. The corresponding three-way interaction contrasts among LESCNet, SA-LSCD, and C3k2-DAPE are positive at +2.70, +9.00, +2.10, and +0.30 points. The negative pairwise terms indicate diminishing average returns when two modules are combined, while the positive three-way terms show that this pairwise pattern changes in the full configuration. Therefore, the data demonstrate non-additivity and context-dependent complementarity, rather than uniform positive pairwise synergy. The complete model still produces the highest mAP point estimates in both tables. Since each cell is a single fixed-split run, the interaction estimates are descriptive, and no statistical significance or general synergy is claimed.

### 4.5. Comparison with Other Detectors

Table 9 and Table 10 report provenance on a method-by-method basis in the Model (result origin) column. Superscript R identifies Faster R-CNN [5], SSD [6], LMSD-YOLO [13], Efficient-YOLO [14], LRTransDet [15], LH-YOLO [17], LSD-DETR [18], AC-YOLO [19], YOLO11n [33], YOLOv10 [34], and RT-DETR-L [35], all reimplemented by us under the common configuration. Superscript P identifies values taken directly from the original Enhanced YOLO [31] and oriented detector [32] papers. Superscript S identifies SLA-YOLO. R and S rows share the same fixed 8:1:1 split, 640 × 640 input, optimizer settings, 400-epoch schedule, preprocessing, checkpoint rule, hardware environment, and evaluation settings. P rows retain the source papers’ non-identical protocols and are interpreted separately.

For R-marked rows, the citations identify the corresponding architectures, and the displayed numerical values were generated in this study. For P-marked rows, the citations are also the numerical sources. Enhanced YOLO’s HRSID values (8.5 M parameters, 19.2 GFLOPs, 90.4% precision, 83.7% recall, 91.0% AP50, 66.7% AP, and 185.2 FPS) are taken directly from [31]. The oriented detector’s SSDD values (95.45% precision, 92.12% recall, and 92.81% AP50) are taken directly from [32] and use native OBB evaluation. Dashes indicate that the corresponding original paper did not report a result for that dataset or metric.

The aggregate values reported in [32] cannot be converted retrospectively into HBB metrics. A geometry-matched evaluation would require the original prediction-level quadrilaterals, conversion by coordinate extrema, preservation of class scores, common HBB NMS, and reevaluation against the same horizontal ground truth. Because those prediction files are not available here, Table 9 retains the published OBB values with superscript c and excludes them from the HBB ranking.

#### 4.5.1. Results on SSDD

On SSDD, SLA-YOLO achieves 97.3% mAP@0.5 and 68.2% mAP@0.5:0.95 with 1.70 million parameters and 3.6 GFLOPs. Compared with the retrained YOLO11n row, these values improve by 4.4 and 12.7 percentage points, respectively. Under the common configuration, SLA-YOLO has the highest mAP@0.5, narrowly exceeding AC-YOLO at 97.2% and LMSD-YOLO at 96.2%. LH-YOLO and LMSD-YOLO obtain higher mAP@0.5:0.95 values of 77.3% and 69.3%, respectively, but require 1.86 million parameters and 9.6 GFLOPs, and 3.50 million parameters and 6.6 GFLOPs. SLA-YOLO provides the smallest parameter count among these three models and substantially lower computation than LH-YOLO. Its network-only throughput is 66.8 FPS, compared with 42.9 FPS for YOLO11n under the identical RTX 3090 protocol.

Within the controlled R- and S-marked SSDD comparison, LMSD-YOLO, Efficient-YOLO, and LH-YOLO obtain 96.2%/69.3%, 92.6%/68.3%, and 94.2%/77.3% for mAP@0.5/mAP@0.5:0.95, respectively. LSD-DETR, LRTransDet, and RT-DETR-L obtain 96.1%/67.6%, 91.3%/66.3%, and 95.3%/66.5%. These results show that SLA-YOLO emphasizes a compact balance rather than leading every accuracy metric. Enhanced YOLO [31] did not report an SSDD result, so that row contains dashes. The original oriented-detector paper [32] reports 95.45% precision, 92.12% recall, and 92.81% AP50 on its 8:2 SSDD split under native OBB evaluation. These P-marked values are included for reference but excluded from the direct HBB ranking because both the data protocol and box geometry differ.

#### 4.5.2. Results on HRSID

On HRSID, SLA-YOLO obtains 90.8% mAP@0.5 and 64.3% mAP@0.5:0.95 with 1.70 million parameters and 3.6 GFLOPs. Relative to YOLO11n, SLA-YOLO gains 5.2 points in mAP@0.5 and 5.3 points in mAP@0.5:0.95. LH-YOLO records the highest mAP@0.5 in the controlled comparison, at 95.1%, while LMSD-YOLO records the highest mAP@0.5:0.95, at 71.1%. Their parameter/GFLOP costs are 1.86 million/9.6 and 3.50 million/6.6, respectively. SLA-YOLO therefore provides lower parameter and computation costs, while its mAP@0.5 is 0.1 point higher than LMSD-YOLO and its mAP@0.5:0.95 is lower. Its network-only throughput is 63.4 FPS, compared with 43.8 FPS for YOLO11n under the identical RTX 3090 protocol.

On HRSID, Efficient-YOLO obtains 86.9%/67.8% mAP@0.5/mAP@0.5:0.95, while LSD-DETR, LRTransDet, RT-DETR-L, and AC-YOLO obtain 90.3%/63.6%, 89.6%/63.8%, 89.7%/61.1%, and 89.3%/67.4%, respectively. The original Enhanced YOLO paper [31] reports 91.0% AP50 and 66.7% AP on HRSID. Although its AP50 is numerically 0.2 points above SLA-YOLO’s 90.8%, Ref. [31] uses its own 65:35 split, 800 × 800 image setting, and source-specific training and evaluation protocol. The difference is therefore descriptive and does not establish a controlled advantage over SLA-YOLO.

The study-generated single-run results support a compact accuracy-complexity operating point under the common training and evaluation configuration. R and S rows in Table 9 and Table 10 belong to this controlled comparison, whereas P rows reproduce original-paper values under non-identical protocols and are interpreted only as contextual references. Transfer with frozen source-trained weights is evaluated separately in Section 4.6, also from single runs. Section 4.7 adds three-seed means, sample standard deviations, and paired contrasts for the principal models. Because each seed also changes the partition, these spreads combine training and split variation and do not isolate either source.

### 4.6. Cross-Dataset Transfer and External Validity

The SSDD and HRSID within-dataset results above used separate training and testing splits. Because these benchmarks share Sentinel-1 and TerraSAR-X source families [3,4], we further evaluated frozen source-trained checkpoints in four no-fine-tuning source → target settings: SSDD → HRSID, HRSID → SSDD, SSDD → LS-SSDD-v1.0, and SSDD → SAR-Ship-Dataset. Each setting compares SLA-YOLO with YOLO11n on the same target data. Table 11 summarizes the two additional target benchmarks.

LS-SSDD-v1.0 is based on 15 large Sentinel-1 scenes and contains 9000 image crops of 800 × 800 pixels with 6015 labeled ships; the original benchmark uses 10 source scenes for training and five for testing [36]. Its small targets, extensive background, and source-scene split make it suitable for examining scale, context, and false-alarm behavior. It does not introduce a new sensor family relative to SSDD or HRSID. SAR-Ship-Dataset combines 102 Gaofen-3 and 108 Sentinel-1 source images. The maintainers’ April 2021 corrected release contains 39,729 image chips of 256 × 256 pixels, with one ship class and horizontal box labels [37]. Its original 2019 paper described an earlier, larger release; all counts here refer to the corrected release. The public repository does not provide verified per-chip sensor provenance for a Gaofen-3-only test, so a sensor-specific result must await provenance checks.

Table 12 reports the completed transfer comparisons using source-trained checkpoints without target-domain fine-tuning or target-based threshold tuning. The SSDD → HRSID and HRSID → SSDD directions test reciprocal weight transfer; the official five-scene LS-SSDD-v1.0 test split (3000 crops) probes large-scene crops and small ships, while SAR-Ship-Dataset probes a different chip collection that includes Gaofen-3 and Sentinel-1 imagery. All evaluations use one ship class and horizontal boxes. For the paired model comparison, preprocessing and metric definitions were held consistent within each target setting.

In the four reported single-run transfer settings, SLA-YOLO has higher mAP@0.5 than YOLO11n: 75.6% versus 69.3% for SSDD → HRSID (+6.3 points), 91.5% versus 86.9% for HRSID → SSDD (+4.6), 69.2% versus 65.7% for SSDD → LS-SSDD-v1.0 (+3.5), and 83.6% versus 79.4% for SSDD → SAR-Ship-Dataset (+4.2). The corresponding observed mAP@0.5:0.95 differences are +2.7, +2.7, +2.8, and +3.4 points, respectively. Precision and recall are also higher for SLA-YOLO in each paired comparison. The direction and target matter: for example, SSDD-trained SLA-YOLO reaches 75.6% mAP@0.5 on HRSID, below its 90.8% within-HRSID result, so the gains over YOLO11n do not imply immunity to dataset shift.

The LS-SSDD-v1.0 result is a crop-level transfer measurement; a full-scene claim would also require tiling, merging, and ship-free-background false-alarm analysis. For SAR-Ship-Dataset, approximately 4000 chips were randomly selected from the corrected release, and 400 of these were used as the target test set (10% of the sampled pool; about 1% of the full release). Some source-scene overlap with the source data remains, and the per-chip sensor composition was not verified. This result is a supplementary chip-level comparison, not an independent-scene or Gaofen-3-only transfer test. Together with the shared sensor families in SSDD and HRSID, these limits confine the conclusion to the tested dataset configurations; unseen-acquisition robustness still requires an independent scene-level test.

### 4.7. Random-Seed and Partition Variability

The study-generated accuracies in Table 1, Table 2, Table 3, Table 4, Table 5, Table 6, Table 7, Table 8, Table 9 and Table 10 and the transfer accuracies in Table 12 derive from individual trained checkpoints and fixed evaluation partitions. An 8:1:1 SSDD division leaves approximately 116 test images. To assess sensitivity to random seed and partition, Table 13 repeats the four principal configurations for seeds 0, 1, and 2 on newly generated 8:1:1 partitions. Models with the same seed share identical train, validation, and test image sets, permitting within-seed paired comparisons. The resulting standard deviations reflect the combined variation from training and image-level partitioning.

For each dataset and configuration, we computed the arithmetic mean and sample standard deviation of the three reported seed-level mAP values. For each adjacent model pair, we subtracted the scores obtained with the same seed and split, then summarized those three paired differences by their mean and sample standard deviation. Summary statistics were calculated before rounding the displayed means and standard deviations. This pairing reduces test-set mismatch within a comparison, whereas changing partitions across seeds captures their contribution to overall variability. It does not separate partition effects from training randomness, and image-level splitting does not guarantee independent SAR source scenes.

Across the three seeded partitions, full SLA-YOLO exceeds YOLO11n by +5.03 ± 0.47 points in mAP@0.5 and +8.73 ± 0.06 points in mAP@0.5:0.95 on SSDD; the corresponding paired HRSID differences are +5.40 ± 0.40 and +6.83 ± 0.64 points. Each full-model difference is positive in all three seeds. Smaller incremental gains vary more relative to their means: for example, the SSDD SA-LSCD addition gives +0.80 ± 0.40 points in mAP@0.5 and +0.97 ± 0.46 in mAP@0.5:0.95. The original fixed-split SSDD full-model localization gain was +12.7 points, whereas the new three-seed paired mean is +8.73 points; the different test partitions explain why the two summaries must be interpreted separately. Three seeds provide an initial variability estimate, not a strong significance test or a guarantee of stable rankings on unseen acquisitions.

### 4.8. Size- and Scene-Stratified Evaluation

The motivation in Section 1 and Section 3 concerns small ships with weak or compact scattering responses and scenes with complex coastal backgrounds. Overall precision, recall, mAP, and selected images do not directly establish performance in these conditions. We therefore conducted a separate quantitative size- and scene-stratified evaluation of YOLO11n and SLA-YOLO on the same seed-specific SSDD and HRSID test partitions used for Table 13. For every seed, both checkpoints used identical preprocessing, confidence threshold, non-maximum suppression, and horizontal-box matching. Table 14 and Table 15 report the mean and sample standard deviation across three independently randomized partitions, together with within-seed paired differences.

For the size analysis, each ship’s horizontal ground-truth box was projected onto the 640 × 640 evaluation canvas using the inference resize rule. Small denotes area < 32^2^ pixels, medium denotes 32^2^ ≤ area < 96^2^ pixels, and large denotes area ≥ 96^2^ pixels. Table 14 reports the observed range of ground-truth ships across the three seed-specific test partitions. AP is averaged over IoU thresholds 0.50:0.05:0.95. These area bins measure projected box size rather than radar backscatter strength, so APsmall provides evidence for small-target detection but does not by itself demonstrate improved detection of weak-scattering ships.

For the scene analysis, an image was treated as inshore when shoreline, land, quay, or port structures were visible, including mixed backgrounds; open-water-only images were treated as offshore. Scene labels were matched to the exact seed-specific test-image lists, and Table 15 reports the observed per-seed ranges of images and ships. Detections and ground truths were evaluated within each image-level group, including false positives in the corresponding background.

SLA-YOLO improves AP in every reported size bin. For small ships, AP increases from 50.8 ± 0.7% to 60.9 ± 0.6% on SSDD and from 54.2 ± 1.0% to 61.1 ± 0.5% on HRSID; the corresponding paired gains are +10.1 ± 0.5 and +6.9 ± 0.7 percentage points. On SSDD, the paired gains decrease to +8.4 ± 0.4 points for medium ships and +5.8 ± 1.4 for large ships. On HRSID, the corresponding medium- and large-ship gains are +6.4 ± 0.5 and +5.6 ± 1.9 points. The largest mean gain therefore occurs in the small bin on both datasets. The large-bin standard deviations are higher, consistent with the much smaller per-seed counts of 20–23 SSDD ships and 18–23 HRSID ships; these large-target results should be interpreted cautiously.

The scene-stratified results show a larger benefit in coastal backgrounds. On SSDD, inshore mAP@0.5/mAP@0.5:0.95 increases from 80.5 ± 2.0%/42.0 ± 1.5% for YOLO11n to 91.0 ± 1.2%/57.0 ± 1.2% for SLA-YOLO, with paired gains of +10.5 ± 1.3 and +15.0 ± 0.9 points. The corresponding offshore gains are +3.7 ± 0.4 and +7.2 ± 0.3 points. On HRSID, inshore performance rises from 61.0 ± 1.4%/37.0 ± 1.6% to 73.0 ± 1.0%/49.0 ± 1.1%, giving paired gains of +12.0 ± 0.8 and +12.0 ± 0.9 points, compared with +3.9 ± 0.4 and +5.7 ± 0.5 points offshore. Absolute inshore accuracy remains lower than offshore accuracy, confirming that coastal clutter is the more difficult condition. These measurements directly support the small-target and complex-coastal-background motivations on the tested partitions. They do not isolate radar scattering strength, which would require a predefined target-contrast or calibrated backscatter analysis.

The inshore/offshore split provides a reproducible scene-context analysis based on visible coastal structures, but it is not a complete ordinal measure of background complexity. We do not report a separate sparse/dense quantitative table because SSDD and HRSID do not provide verified density labels and no density threshold was defined before this analysis. Dense and sparse examples are retained in Section 4.9 as qualitative illustrations only; they are not used as substitutes for stratified metrics. A future density analysis should preregister a definition based on ships per image or nearest-neighbor spacing and evaluate it on fixed image lists.

### 4.9. Qualitative Analysis

**Figure 7:**
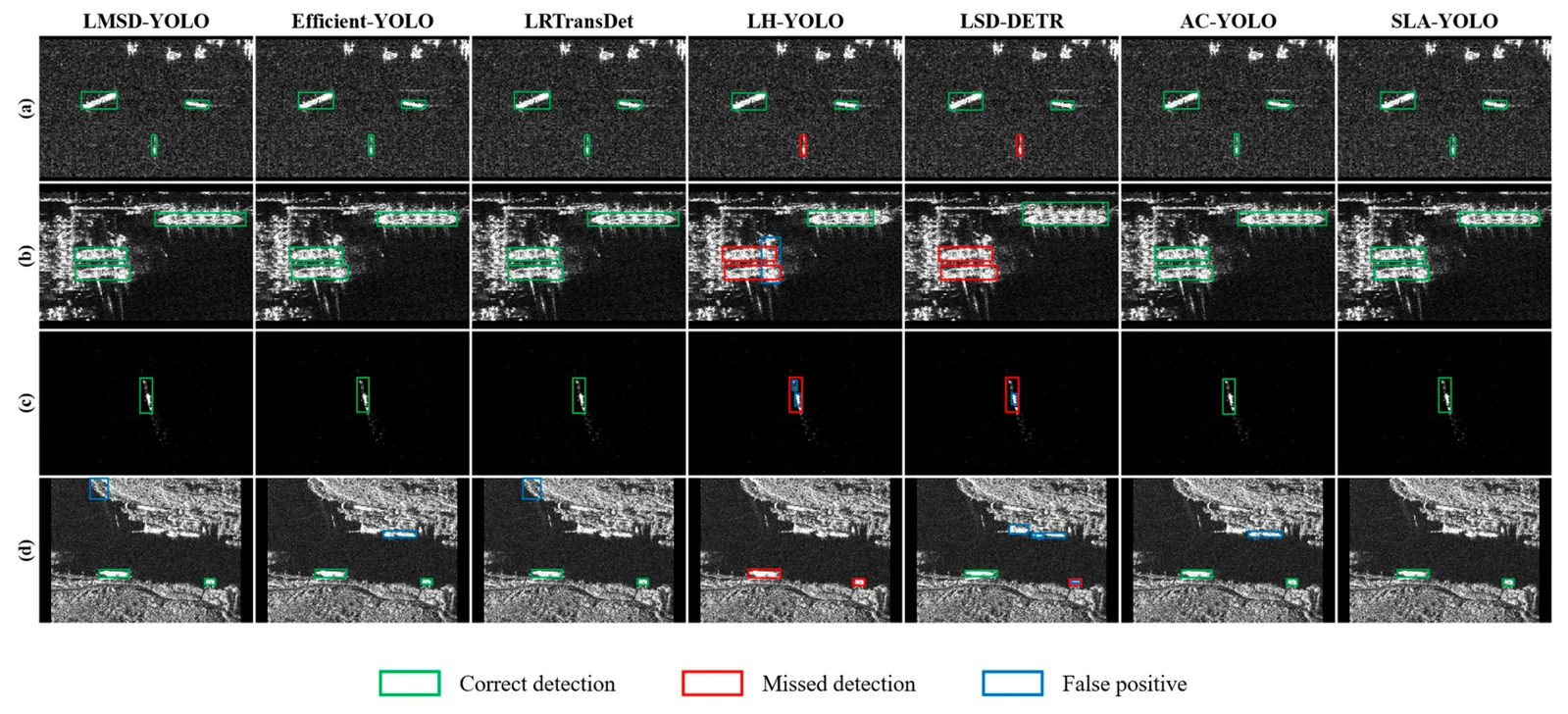
Qualitative detection results on SSDD. Rows show (**a**) sparse offshore ships, (**b**) densely berthed ships in a coastal scene, (**c**) an isolated weak target, and (**d**) ships in a complex inshore scene.

**Figure 8:**
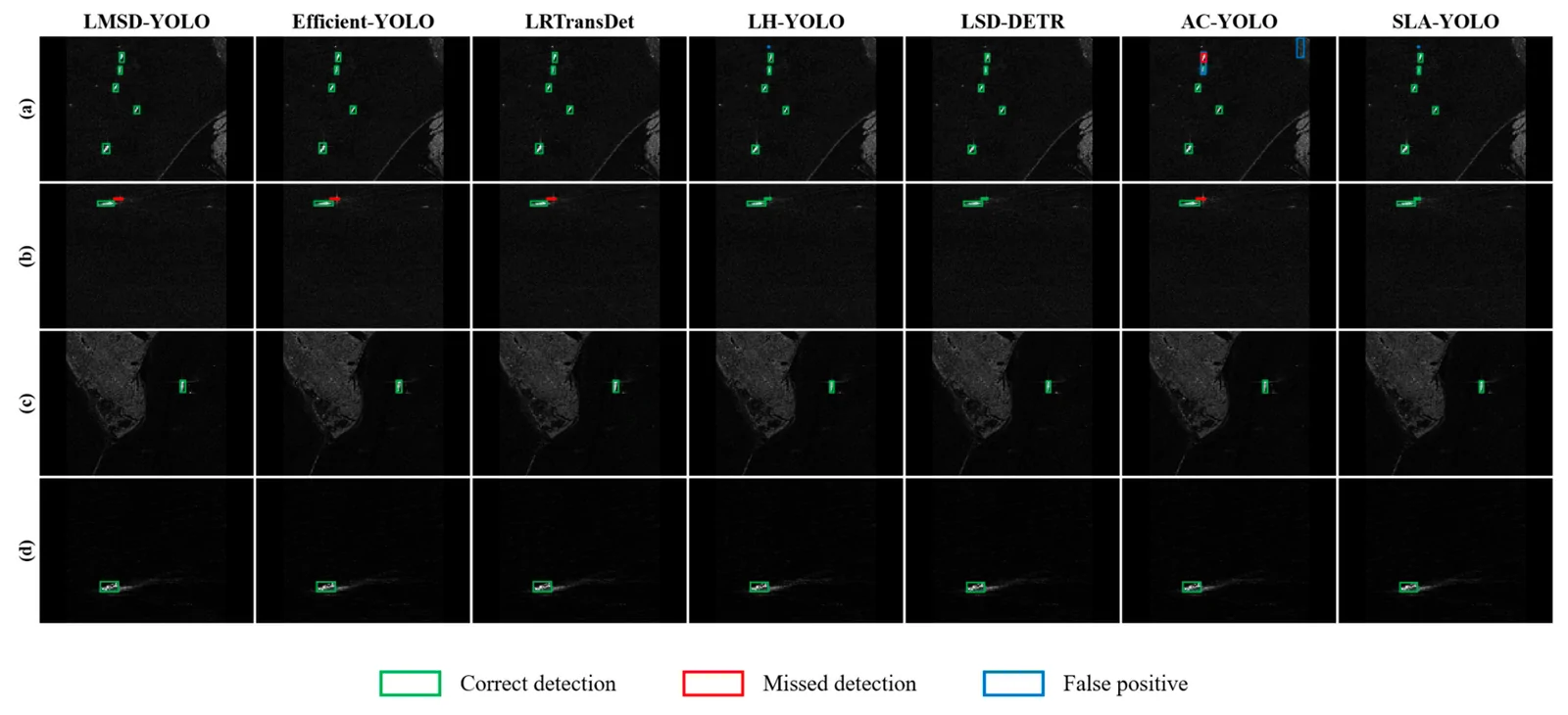
Qualitative detection results on HRSID. Rows show (**a**) multiple small ships near a coastal boundary, (**b**) adjacent low-contrast ships near the upper image boundary, (**c**) an isolated ship beside coastal land, and (**d**) an isolated ship against a dark-sea background.

Figure 7 and Figure 8 retain the available same-scene qualitative comparisons for LMSD-YOLO, Efficient-YOLO, LRTransDet, LH-YOLO, LSD-DETR, AC-YOLO, and SLA-YOLO. The displayed predictions were generated from our reimplementations on the same example images and under the same experimental configuration used for the quantitative comparisons. LMSD-YOLO, Efficient-YOLO, and LH-YOLO are now included in both the quantitative comparisons in Table 9 and Table 10 and the qualitative panels. Green, red, and blue boxes denote correct detections, missed detections, and false positives. The figures are visual illustrations and are not used to infer or replace quantitative metrics.

In the SSDD examples, SLA-YOLO detects the complete set of small separated ships in scene (a) and avoids the missed detections produced by LH-YOLO and LSD-DETR. In the dense coastal scene (b), several competing methods merge or miss adjacent vessels, whereas SLA-YOLO retains distinct boxes. In scene (c), the proposed method localizes the isolated weak target without the false-positive behavior visible in some alternatives. In the complex inshore scene (d), it avoids both the false alarms and missed detections observed in the comparison methods.

The HRSID examples show a similar qualitative pattern. The model preserves multiple small targets in a sparse scene, detects a low-contrast vessel near the upper boundary, and maintains stable boxes in examples containing dark sea backgrounds and coastal structures. These observations are consistent with the size- and scene-stratified results in Section 4.8, but the selected images do not isolate the contribution of each component.

The selected examples provide interpretable evidence for the depicted scenes only. Although the models were reimplemented under the same configuration, selected qualitative panels do not establish a general superiority claim. Section 4.6 supplies separate cross-dataset measurements; Section 4.8 provides controlled size and scene comparisons on the tested partitions; sensor-resolved analysis would further clarify transfer to new acquisitions.

### 4.10. Deployment-Oriented Analysis

The complete SLA-YOLO model contains 1.70 million parameters. Assuming 32-bit floating-point storage, the raw weights require approximately 6.8 MB before framework overhead. Lower-precision inference could reduce storage further, although quantization was not evaluated in this study. The 3.6 GFLOP workload is 42.9% lower than the YOLO11n baseline. Together with the controlled RTX 3090 throughput measurements, these results demonstrate algorithmic compactness and efficient desktop-GPU execution under the stated network-only protocol.

On the RTX 3090, the SSDD- and HRSID-trained SLA-YOLO checkpoints achieve 66.8 and 63.4 network-only FPS, respectively, whereas the corresponding YOLO11n checkpoints achieve 42.9 and 43.8 FPS. These values were obtained at batch size 1, 640 × 640, and FP32 after 100 warm-up iterations and over 500 CUDA-synchronized timed iterations, excluding preprocessing, host-to-device transfer, and NMS. They demonstrate higher network-only throughput than YOLO11n on the tested desktop GPU. Because operator support, memory bandwidth, power mode, and runtime optimization differ across platforms, these measurements should not be extrapolated directly to satellite processors, FPGAs, mobile GPUs, CPUs, or Jetson devices.

An NVIDIA Jetson or comparable embedded platform was not available for this study, so we cannot yet report a defensible embedded-device latency or energy measurement. This hardware limitation is stated explicitly rather than treating the RTX 3090 result as on-board validation. The next deployment stage will export the model to a standardized inference format and benchmark it on representative embedded hardware under fixed batch size, resolution, precision, warm-up, timed-run count, synchronization, preprocessing, data-transfer, and NMS settings. Operator profiling, mixed-precision or integer quantization, power measurement, and end-to-end tests including image tiling and georeferencing will then determine whether the desktop-GPU advantage transfers to the intended platform.

## 5. Conclusions

This study presents SLA-YOLO, a lightweight attention-enhanced network for multiscale ship detection in SAR imagery. The model coordinates three components. LESCNet uses ELDWConv and RSCB to provide efficient spatial-channel representation; C3k2-DAPE performs input-adaptive four-branch fusion followed by channel refinement and residual integration; and SA-LSCD shares convolutional parameters across three scales while preserving independent outputs and applying channel-spatial attention.

In the original fixed-split SSDD and HRSID experiments, SLA-YOLO achieves mAP@0.5 values of 97.3% and 90.8% and mAP@0.5:0.95 values of 68.2% and 64.3%, respectively. Compared with YOLO11n, parameters and GFLOPs are reduced by 34.1% and 42.9%, while mAP@0.5:0.95 improves by 12.7 and 5.3 percentage points under the original fixed-split protocol. The complete 2^3^ factorial ablation includes all standalone, pairwise, and three-component configurations. All three components have positive average main effects on both mAP measures and datasets, and the complete model has the highest mAP point estimates. The negative pairwise and positive three-way interaction contrasts indicate non-additive, context-dependent returns rather than uniform pairwise synergy. Because the eight factorial cells are single fixed-split runs, repeated runs across all cells are still required for inferential interaction claims; operator-level attribution and scene-dependent branch specialization also require mechanism-level ablations and branch-weight analysis. Under the specified batch-1 FP32 network-only protocol, the SSDD- and HRSID-trained checkpoints achieve 66.8 and 63.4 FPS on the RTX 3090, compared with 42.9 and 43.8 FPS for YOLO11n; embedded-device latency and energy remain to be evaluated on representative hardware. The 42.9% GFLOPs reduction is therefore interpreted only as a reduction in arithmetic workload; it is not used to infer latency, and the throughput claim rests on the separately measured RTX 3090 FPS values under the stated protocol. Across four no-fine-tuning source-to-target evaluations, SLA-YOLO improves mAP@0.5 over YOLO11n by 3.5–6.3 points and mAP@0.5:0.95 by 2.7–3.4 points. The best evidence is for the specified benchmark protocols; the 400-chip SAR-Ship-Dataset test contains some scene overlap with source data. Large-scene false alarms, per-sensor transfer, and unseen-acquisition robustness require additional analysis. The three-seed analysis in Table 13 yields SLA-YOLO mean ± SD mAP@0.5:0.95 of 67.20 ± 0.40% on SSDD and 64.23 ± 0.35% on HRSID. Its paired gains over YOLO11n are +8.73 ± 0.06 and +6.83 ± 0.64 points, respectively; all three recorded seeds favor the full model. The single-run and repeated-seed results use different image partitions, and the three-seed sample does not establish statistical significance for small component increments. Further source-scene-controlled testing is needed to determine robustness to unseen acquisitions. The size-stratified results show small-ship AP gains of +10.1 ± 0.5 points on SSDD and +6.9 ± 0.7 points on HRSID. Inshore mAP@0.5:0.95 gains of +15.0 ± 0.9 and +12.0 ± 0.9 points exceed the corresponding offshore gains of +7.2 ± 0.3 and +5.7 ± 0.5 points. These comparisons support improved performance for small projected targets and complex coastal backgrounds on the tested partitions. Because box area and shoreline context do not measure radar backscatter, they do not isolate weak-scattering performance.

## Figures and Tables

**Table 1:** Backbone-stage ablation results on SSDD with the baseline neck and head retained. ELDWConv denotes the LESCNet-only detector rather than the complete SLA-YOLO model. The table contains single-run results on the fixed SSDD split.

Conv	GSConv	ELDWConv	Params (M)	GFLOPs	P (%)	R (%)	mAP@0.5 (%)	mAP@0.5:0.95 (%)
			2.58	6.3	93.1	88.4	92.9	55.5
√			1.94	5.0	94.2	92.2	96.8	65.5
	√		1.68	4.5	93.2	92.7	96.7	64.4
		√	1.86	4.3	95.2	91.9	96.8	65.6

**Table 2:** Backbone-stage ablation results on HRSID with the baseline neck and head retained. In this table, the ELDWConv row again refers to the LESCNet-only detector rather than the full model. The table contains single-run results on the fixed HRSID split.

Conv	GSConv	ELDWConv	Params (M)	GFLOPs	P (%)	R (%)	mAP@0.5 (%)	mAP@0.5:0.95 (%)
			2.58	6.3	88.8	75.4	85.6	59.0
√			1.94	5.0	89.4	79.5	89.8	60.0
	√		1.68	4.5	90.2	76.6	88.0	61.6
		√	1.86	4.3	87.9	80.2	89.1	61.8

**Table 3:** Detection-head ablation results on SSDD with LESCNet fixed. LSCD + ASFF is the ASFF-based comparison head, whereas SA-LSCD is the proposed CBAM-based shared head. The reported values are from one fixed-split run.

LESCNet	LSCD + ASFF	SA-LSCD	Params (M)	GFLOPs	P (%)	R (%)	mAP@0.5 (%)	mAP@0.5:0.95 (%)
√			1.86	4.3	95.2	91.9	96.8	65.6
√	√		1.73	3.8	96.0	92.5	96.7	65.0
√		√	1.70	3.6	96.1	91.8	96.5	65.9

**Table 4:** Detection-head ablation results on HRSID with LESCNet fixed. The ASFF comparison head is labeled LSCD + ASFF, while SA-LSCD denotes the proposed CBAM-based shared head. The reported values are from one fixed-split run.

LESCNet	LSCD + ASFF	SA-LSCD	Params (M)	GFLOPs	P (%)	R (%)	mAP@0.5 (%)	mAP@0.5:0.95 (%)
√			1.86	4.3	87.9	80.2	89.1	61.8
√	√		1.73	3.8	90.4	81.8	90.7	61.9
√		√	1.70	3.6	91.5	80.3	90.6	64.2

**Table 5:** Neck-stage ablation results for C3k2-AP and C3k2-DAPE on SSDD with LESCNet and SA-LSCD fixed. The values correspond to one run on the fixed SSDD split.

LESCNet	SA-LSCD	C3k2-AP	C3k2-DAPE	Params (M)	GFLOPs	P (%)	R (%)	mAP@0.5 (%)	mAP@0.5:0.95 (%)
√	√			1.70	3.6	96.1	91.8	96.5	65.9
√	√	√		1.61	3.6	98.1	91.2	96.8	65.6
√	√		√	1.70	3.6	97.9	90.8	97.3	68.2

**Table 6:** Neck-stage ablation results for C3k2-AP and C3k2-DAPE on HRSID with LESCNet and SA-LSCD fixed. Params and GFLOPs are reported for every row, and the values correspond to one run on the fixed HRSID split.

LESCNet	SA-LSCD	C3k2-AP	C3k2-DAPE	Params (M)	GFLOPs	P (%)	R (%)	mAP@0.5 (%)	mAP@0.5:0.95 (%)
√	√			1.70	3.6	91.5	80.3	90.6	64.2
√	√	√		1.61	3.6	91.7	80.2	90.6	63.9
√	√		√	1.70	3.6	88.6	82.8	90.8	64.3

**Table 7:** Complete 2^3^ factorial ablation results on SSDD for LESCNet, SA-LSCD, and C3k2-DAPE. All eight configurations use the same fixed SSDD split and training protocol; Section 4.7 reports three-seed results for the four progressive configurations.

LESCNet	SA-LSCD	C3k2-DAPE	Params (M)	GFLOPs	P (%)	R (%)	mAP@0.5 (%)	mAP@0.5:0.95 (%)
			2.58	6.3	93.1	88.4	92.9	55.5
√			1.86	4.3	95.2	91.9	96.8	65.6
	√		1.83	4.2	95.6	91.3	95.3	64.6
		√	1.85	4.6	95.5	91.5	95.8	65.1
√	√		1.70	3.6	96.1	91.8	96.5	65.9
√		√	1.71	3.8	96.3	91.3	96.8	66.8
	√	√	1.72	3.7	96.5	91.5	96.3	66.3
√	√	√	1.70	3.6	97.9	90.8	97.3	68.2

**Table 8:** Complete 2^3^ factorial ablation results on HRSID for LESCNet, SA-LSCD, and C3k2-DAPE. All eight configurations use the same fixed HRSID split and training protocol; Section 4.7 reports three-seed results for the four progressive configurations.

LESCNet	SA-LSCD	C3k2-DAPE	Params (M)	GFLOPs	P (%)	R (%)	mAP@0.5 (%)	mAP@0.5:0.95 (%)
			2.58	6.3	88.8	75.4	85.6	59.0
√			1.86	4.3	87.9	80.2	89.1	61.8
	√		1.83	4.2	89.3	79.4	89.3	62.3
		√	1.85	4.6	89.6	79.1	88.9	62.5
√	√		1.70	3.6	91.5	80.3	90.6	64.2
√		√	1.71	3.8	90.3	81.1	89.6	63.5
	√	√	1.72	3.7	89.6	80.9	90.2	63.9
√	√	√	1.70	3.6	88.6	82.8	90.8	64.3

**Table 9:** Comparison on SSDD with method-specific result origin. Superscripts R, P, and S identify author-reimplemented methods, original-paper values, and SLA-YOLO, respectively.

Model (Result Origin)	Params (M)	GFLOPs	P (%)	R (%)	mAP@0.5 (%)	mAP@0.5:0.95 (%)	FPS (f/s)
AC-YOLO [19] ^R^	1.816	5.4	96.1	91.4	97.2	62.9	60.38
Enhanced YOLO [31] ^P^	—	—	—	—	—	—	—
Oriented detector [32] ^P^	—	—	95.45 ^c^	92.12 ^c^	92.81 ^c^	—	—
Faster R-CNN [5] ^R^	41.36	83.6	77.5	81.3	93.9	62.1	68.0
SSD [6] ^R^	23.75	30.4	62.8	69.3	90.2	57.9	56.3
LMSD-YOLO [13] ^R^	3.50	6.6	96.7	93.4	96.2	69.3	60.3
Efficient-YOLO [14] ^R^	8.2	3.8	96.2	84.2	92.6	68.3	63.0
LH-YOLO [17] ^R^	1.86	9.6	90.5	88.9	94.2	77.3	41.6
YOLOv10 [34] ^R^	8.04	24.8	89.0	86.6	92.7	58.7	50.6
YOLO11n [33] ^R^	2.58	6.3	93.1	88.4	92.9	55.5	42.9
RT-DETR-L [35] ^R^	41.94	125.6	93.5	93.0	95.3	66.5	69.3
LRTransDet [15] ^R^	3.07	3.85	86.4	88.1	91.3	66.3	63.2
LSD-DETR [18] ^R^	8.8	—	94.3	91.4	96.1	67.6	35.6
SLA-YOLO ^S^	1.70	3.6	97.9	90.8	97.3	68.2	66.8

Note: R identifies a competing method reimplemented by the authors under the common configuration; P identifies a value taken directly from the original paper under that paper’s protocol; S identifies SLA-YOLO. Dashes denote values not reported by the source. For [32], superscript c marks published native-OBB precision, recall, and AP50. These values use an 8:2 SSDD split, 36 training epochs, and rotated geometry and are not directly comparable with the HBB metrics from the R and S rows. FPS for R and S uses batch size 1, 640 × 640 input, FP32, 100 warm-up iterations, and 500 CUDA-synchronized network-only timed iterations; preprocessing, host-to-device transfer, and NMS are excluded.

**Table 10:** Comparison of HRSID with method-specific result origin. The R, P, and S superscripts distinguish author-reimplemented methods, values copied from the cited papers, and SLA-YOLO, respectively.

Model (Result Origin)	Params (M)	GFLOPs	P (%)	R (%)	mAP@0.5 (%)	mAP@0.5:0.95 (%)	FPS (f/s)
AC-YOLO [19] ^R^	1.816	5.4	88.1	82.3	89.3	67.4	50.9
Enhanced YOLO [31] ^P^	8.5	19.2	90.4	83.7	91.0	66.7	185.2
Oriented detector [32] ^P^	—	—	—	—	—	—	—
Faster R-CNN [5] ^R^	41.36	83.6	78.8	82.1	87.3	61.2	41.3
SSD [6] ^R^	23.75	30.4	67.9	73.7	63.3	46.7	54.2
LMSD-YOLO [13] ^R^	3.5	6.6	92.6	85.7	90.7	71.1	50.9
Efficient-YOLO [14] ^R^	8.2	3.8	93.1	71.3	86.9	67.8	41.3
LH-YOLO [17] ^R^	1.86	9.6	92.6	88.4	95.1	69.7	50.3
YOLOv10 [34] ^R^	8.04	24.8	92.3	75.6	87.1	62.4	65.3
YOLO11n [33] ^R^	2.58	6.3	88.8	75.4	85.6	59.0	43.8
RT-DETR-L [35] ^R^	41.94	125.6	93.1	83.6	89.7	61.1	65.4
LRTransDet [15] ^R^	3.07	3.85	85.1	85.3	89.6	63.8	63.7
LSD-DETR [18] ^R^	8.8	—	89.3	86.5	90.3	63.6	22.0
SLA-YOLO ^S^	1.70	3.6	88.6	82.8	90.8	64.3	63.4

Note: In Table 10, R marks a competing method reimplemented by the authors under the common configuration, P marks a value copied from the cited paper under its original protocol, and S marks SLA-YOLO. Dashes denote values not reported by the source. The Enhanced YOLO row is copied from [31], which uses the original HRSID 65:35 training-test division and 800 × 800 image chips; its FPS also retains the source paper’s hardware and timing conditions. The oriented detector [32] did not report HRSID results. Therefore, P-marked values are contextual and are not treated as controlled comparisons with the R and S rows.

**Table 11:** Published characteristics of the additional transfer-evaluation benchmarks. Dataset descriptors are distinct from the results in Table 12.

Benchmark	Source Imagery	Published Size	Role in Transfer Analysis
LS-SSDD-v1.0 [36]	Sentinel-1; 15 large scenes	9000 crops (800 × 800); 6015 ships	Large-scene, small-ship, and background shift
SAR-Ship-Dataset [37]	102 Gaofen-3 + 108 Sentinel-1 source images	39,729 chips (256 × 256); corrected 2021 release	Chip appearance; sensor shift only after chip provenance audit

**Table 12:** Single-run cross-dataset performance without target-domain fine-tuning. All values are percentages; multi-seed variability has not been measured.

Train → Test	Model	P (%)	R (%)	mAP@0.5 (%)	mAP@0.5:0.95 (%)
SSDD → HRSID	YOLO11n	80.8	60.6	69.3	52.2
SSDD → HRSID	SLA-YOLO	81.4	65.1	75.6	54.9
HRSID → SSDD	YOLO11n	88.5	83.1	86.9	55.6
HRSID → SSDD	SLA-YOLO	93.7	85.6	91.5	58.3
SSDD → LS-SSDD-v1.0	YOLO11n	70.8	60.8	65.7	45.4
SSDD → LS-SSDD-v1.0	SLA-YOLO	74.6	62.5	69.2	48.2
SSDD → SAR-Ship-Dataset	YOLO11n	81.7	72.6	79.4	49.5
SSDD → SAR-Ship-Dataset	SLA-YOLO	84.9	74.2	83.6	52.9

**Table 13:** Three-seed accuracy and paired adjacent-stage differences on SSDD and HRSID. Values are mean ± sample SD across seeds 0, 1, and 2. The 8:1:1 partition changes by seed; models within a seed use the same images. Accuracy values are percentages, and Δ values are percentage points.

Dataset	Configuration	mAP@0.5 Mean ± SD (%)	mAP@0.5:0.95 Mean ± SD (%)	Paired ΔmAP@0.5 Mean ± SD (pp)	Paired ΔmAP@0.5:0.95 Mean ± SD (pp)
SSDD	YOLO11n	92.13 ± 0.57	58.47 ± 0.35	—	—
SSDD	LESCNet	95.53 ± 0.40	64.60 ± 0.50	+3.40 ± 0.66	+6.13 ± 0.15
SSDD	LESCNet + SA-LSCD	96.33 ± 0.35	65.57 ± 0.25	+0.80 ± 0.40	+0.97 ± 0.46
SSDD	SLA-YOLO	97.17 ± 0.31	67.20 ± 0.40	+0.83 ± 0.40	+1.63 ± 0.38
HRSID	YOLO11n	85.47 ± 0.35	57.40 ± 0.96	—	—
HRSID	LESCNet	86.30 ± 0.40	61.47 ± 0.35	+0.83 ± 0.75	+4.07 ± 0.67
HRSID	LESCNet + SA-LSCD	89.77 ± 0.38	63.03 ± 0.60	+3.47 ± 0.25	+1.57 ± 0.25
HRSID	SLA-YOLO	90.87 ± 0.35	64.23 ± 0.35	+1.10 ± 0.46	+1.20 ± 0.26

**Table 14:** Measured size-stratified HBB AP on SSDD and HRSID. AP values are mean ± sample SD (%) over three seed-specific test partitions; paired Δ values are percentage points. Ground-truth counts are the observed per-seed range.

Dataset	Size Bin	GT Ships/Seed (Range)	YOLO11n AP Mean ± SD (%)	SLA-YOLO AP Mean ± SD (%)	Paired ΔAP Mean ± SD (pp)
SSDD	Small	88–112	50.8 ± 0.7	60.9 ± 0.6	+10.1 ± 0.5
SSDD	Medium	95–121	64.2 ± 0.5	72.6 ± 0.5	+8.4 ± 0.4
SSDD	Large	20–23	67.9 ± 2.4	73.7 ± 2.1	+5.8 ± 1.4
HRSID	Small	1170–1494	54.2 ± 1.0	61.1 ± 0.5	+6.9 ± 0.7
HRSID	Medium	438–449	66.4 ± 0.9	72.8 ± 0.6	+6.4 ± 0.5
HRSID	Large	18–23	69.0 ± 4.3	74.6 ± 3.8	+5.6 ± 1.9

**Table 15:** Measured inshore/offshore HBB evaluation on SSDD and HRSID. Each mAP cell reports mAP@0.5/mAP@0.5:0.95 as mean ± sample SD (%) over three seed-specific test partitions; paired Δ values are percentage points. Counts are the observed per-seed range.

Dataset	Scene	Images/Ships per Seed	YOLO11n mAP@0.5 mAP@0.5:0.95 (%)	SLA-YOLO mAP@0.5 mAP@0.5:0.95 (%)	Paired Δ mAP@0.5 mAP@0.5:0.95 (pp)
SSDD	Inshore	20–25 img; 75–94 ships	80.5 ± 2.0/ 42.0 ± 1.5	91.0 ± 1.2/ 57.0 ± 1.2	+10.5 ± 1.3/ +15.0 ± 0.9
SSDD	Offshore	91–96 img; 169–181 ship	95.0 ± 0.4/ 62.5 ± 0.5	98.7 ± 0.2/ 69.7 ± 0.4	+3.7 ± 0.4/ +7.2 ± 0.3
HRSID	Inshore	94–111 img; 820–1010 ship	61.0 ± 1.4/ 37.0 ± 1.6	73.0 ± 1.0/ 49.0 ± 1.1	+12.0 ± 0.8/ +12.0 ± 0.9
HRSID	Offshore	449–466 img; 720–850 ship	91.0 ± 0.4/ 62.0 ± 0.6	94.9 ± 0.3/ 67.7 ± 0.4	+3.9 ± 0.4/ +5.7 ± 0.5

## Data Availability

The four public datasets used for the reported experiments are SSDD (https://github.com/TianwenZhang0825/Official-SSDD, accessed on 24 September 2026), HRSID (https://github.com/chaozhong2010/HRSID, accessed on 24 September 2026), LS-SSDD-v1.0 (https://github.com/TianwenZhang0825/LS-SSDD-v1.0-OPEN, accessed on 24 September 2026), and the April 2021 corrected SAR-Ship-Dataset release (https://github.com/CAESAR-Radi/SAR-Ship-Dataset, accessed on 24 September 2026). The fixed train, validation, and test image lists used for the study-generated single-run comparisons, the seed-specific lists for seeds 0, 1, and 2, source-scene identifiers where available, corresponding configuration files, and implementation code will be released with the final manuscript to support replication.
